# Biological Soil Crusts of Arctic Svalbard—Water Availability as Potential Controlling Factor for Microalgal Biodiversity

**DOI:** 10.3389/fmicb.2017.01485

**Published:** 2017-08-08

**Authors:** Nadine Borchhardt, Christel Baum, Tatiana Mikhailyuk, Ulf Karsten

**Affiliations:** ^1^Applied Ecology and Phycology, Institute of Biological Sciences, University of Rostock Rostock, Germany; ^2^Soil Science, Faculty of Agricultural and Environmental Sciences, University of Rostock Rostock, Germany; ^3^Department of Phycology, Lichenology and Bryology, M.H. Kholodny Institute of Botany, National Academy of Sciences of Ukraine Kyiv, Ukraine

**Keywords:** chlorophyta, streptophyta, ochrophyta, precipitation, pH-value, soil properties, Spitsbergen

## Abstract

In the present study the biodiversity of biological soil crusts (BSCs) formed by phototrophic organisms were investigated on Arctic Svalbard (Norway). These communities exert several important ecological functions and constitute a significant part of vegetation at high latitudes. Non-diatom eukaryotic microalgal species of BSCs from 20 sampling stations around Ny-Ålesund and Longyearbyen were identified by morphology using light microscopy, and the results revealed a high species richness with 102 species in total. 67 taxa belonged to Chlorophyta (31 Chlorophyceae and 36 Trebouxiophyceae), 13 species were Streptophyta (11 Klebsormidiophyceae and two Zygnematophyceae) and 22 species were Ochrophyta (two Eustigmatophyceae and 20 Xanthophyceae). Surprisingly, *Klebsormidium* strains belonging to clade G (Streptophyta), which were so far described from Southern Africa, could be determined at 5 sampling stations. Furthermore, comparative analyses of Arctic and Antarctic BSCs were undertaken to outline differences in species composition. In addition, a pedological analysis of BSC samples included C, N, S, TP (total phosphorus), and pH measurements to investigate the influence of soil properties on species composition. No significant correlation with these chemical soil parameters was confirmed but the results indicated that pH might affect the BSCs. In addition, a statistically significant influence of precipitation on species composition was determined. Consequently, water availability was identified as one key driver for BSC biodiversity in Arctic regions.

## Introduction

Biological soil crusts (BSCs) represent a community of various organisms associated with soil particles within and on top of the upper few millimeters of the soil. Algae, cyanobacteria, lichens, bacteria, microfungi, and bryophytes in different proportions form a thin layer on the soil surface, where filamentous algae, and cyanobacteria stick together with soil particles by their mucilaginous sheaths and excreted extracellular polymeric substances (EPS) (Belnap et al., 2001). These EPS consist of sticky polysaccharides and proteins, and are typically released by abundant BSC green algae, such as members of the genera *Coccomyxa* and *Klebsormidium*, which leads to adhesion to soil particles (Barberousse et al., 2006). Some of the EPS adhesives exhibit strong intrinsic mechanical properties (Mostaert et al., 2009), and their producers were frequently observed in BSCs of Antarctica (Pfaff et al., 2016).

The composition and development of BSCs can be very different and hence, various types are distinguished by macroscopic as well as microscopic characteristics (Belnap et al., 2001; Büdel et al., 2009; Williams et al., 2016). BSC types of Arctic Svalbard and Livingston Island, Antarctica were recently described based on visible features, such as the presence/absence of functional groups, their dominance and topography (Williams et al., 2016). The establishment and development of these cryptogamic communities is influenced by several biotic as well as abiotic parameters like pedological properties, climatic factors, and intervention by animals and humans (Elster et al., 1999; Büdel et al., 2009; Langhans et al., 2009; Pushkareva et al., 2016). BSCs are pioneer communities and have important ecological functions in soil stabilization against water and wind erosion (Van Den Ancker and Jungerius, 1985; Eldridge and Greene, 1994; Belnap and Gillette, 1998), change in hydrology as water retention (reviewed in Belnap, 2006; Breen and Lévesque, 2008), primary production and nitrogen fixation (Evans and Lange, 2001; Belnap, 2002; Bhatnagar et al., 2008; Zhang et al., 2009), biogeochemistry as well as geomorphology (Evans and Belnap, 1999) and in nutrient cycles (Wu et al., 2013; Baumann et al., 2017). Furthermore, BSCs have positive influence on the seed germination and plant growth because they enrich nutrients in the soil (Belnap et al., 2001; Evans and Lange, 2001; Harper and Belnap, 2001; Belnap, 2003; Breen and Lévesque, 2008; Ghiloufi et al., 2016).

BSCs are distributed worldwide in all climatic zones and occur mostly in extreme and nutrient-poor habitats, such as hot and cold, semiarid and arid areas, and can be the only vegetation in these landscapes (Belnap and Lange, 2003). Colesie et al. (2014) reported BSCs in continental Antarctica and Borchhardt et al. (2017) in maritime Antarctica. The latter authors also provided for the first time a comprehensive species list of microalgae and lichens of BSCs. Furthermore, a comparative geo-ecological study described various BSC types of Antarctic Livingston Island and Arctic Svalbard, but without any information on biodiversity (Williams et al., 2016). BSCs are generally poorly studied in the Polar Regions until now (Green and Broady, 2001), and a recent review by Pushkareva et al. (2016) summarized all information on Arctic BSCs and concluded that much more studies on the biodiversity of BSCs at high latitudes are needed because of ongoing climate change. In addition, a considerable high areal coverage of BSCs (up to 90%) on Svalbard was recently reported by Williams et al. (2016) which indicated that BSCs might be the prevailing vegetation type at such high latitudes. It is assumed that the BSC composition as well as their distribution will shift or BSCs will even be displaced by invasive species due to climate change in the Polar Regions (Frenot et al., 2005; Pushkareva et al., 2016). Consequently, deeper investigations on the biodiversity of Polar BSCs are urgently required and will enable better prediction of future vegetation development at high latitudes.

Therefore, in the present study two major scientific questions were addressed. Firstly, the microalgal diversity of Arctic BSCs was investigated. Secondly, local habitat differences were analyzed with a major focus on soil characteristics and precipitation, as these factors might have a strong effect on microalgal species composition in BSCs.

## Materials and methods

### Study sites

**Figure 1:**
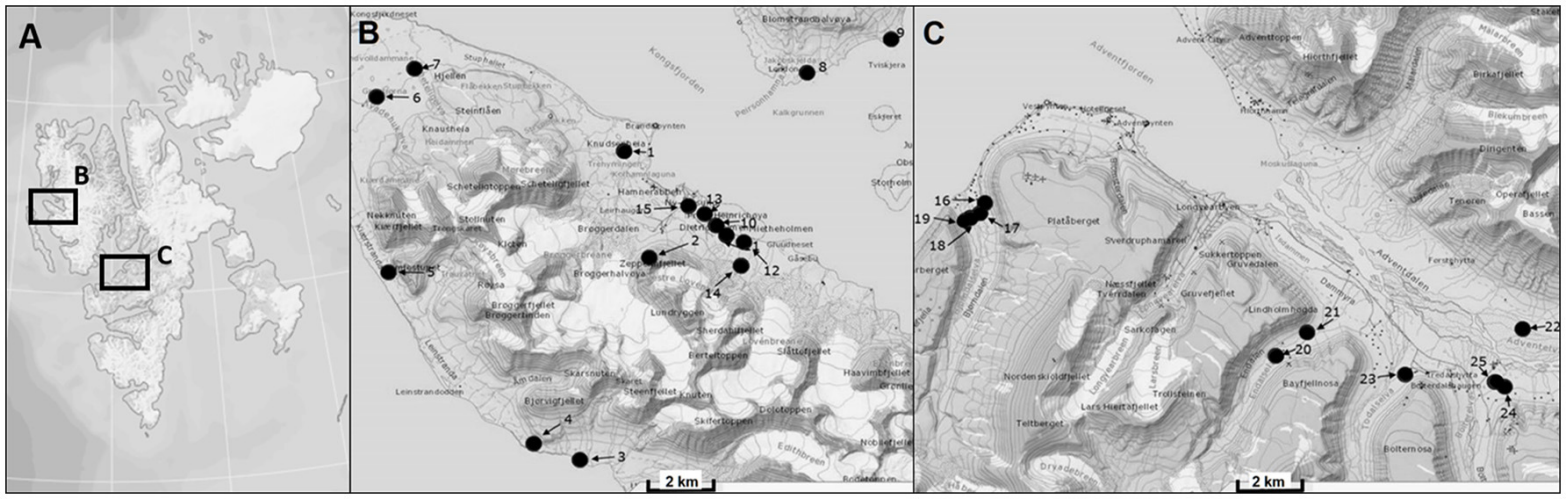
Map of sampling areas for collecting biological soil crusts. **(A)** The two investigated sampling localities on Svalbard, **(B)** sampling area around Ny-Ålesund, **(C)** sampling area around Longyearbyen. Numbers of sampling stations are explained in Table 1.

Svalbard is an archipelago located in the North Atlantic sector of the Arctic Ocean which ranges from 74° to 81° north latitude and from 10° to 35° east longitude, and the capital Longyearbyen is administered by Norway. This group of islands has a mild climate compared to regions at the same latitudes because the West Spitsbergen Current (WSC) transports warm Atlantic water masses into the Arctic Ocean along the West coast of Svalbard. The expedition took place in August 2014 and BSC samples were collected at 20 sampling stations around the two research areas Ny-Ålesund (78°55′26.33″N, 11°550′23.84″E) and Longyearbyen (78°13′10.18″N, 15°39′7.19″E) (Figure 1, Table 1). The mean temperature in summer is 8°C for Ny-Ålesund and 5°C for Longyearbyen and −14°C (maximum −35°C) for both localities in winter. The annual precipitation differs between both sampling areas, with an average of 471 mm in Ny-Ålesund and a frequency of 198 precipitation days, while Longyearbyen with 205 mm rain and snow fall at 199 days is much drier. About 70% of precipitation typically falls between October and May, when these areas are usually completely covered by snow (Norwegian Meteorological Institute)^1^.

**Table 1:** Sampling stations on Arctic Svalbard.

**Station number**	**Station name**	**Abbreviation**	**Coordinates**
1	Brandal foreland	BV	78°56.285″N 11°49.769″E
3	Daerten	Dae	78°51.009″N 11°47.532″E
4	between Daerten and Stenahytten	D-S	78°51.240″N 11°43.571″E
5	between Stenahytten and Kjsvika	SH-Kj	78°54.009″N 11°30.550″E
6	Geopol	Geo	78°56.973″N 11°28.594″E
7	between Geopol and Kongsfjorden coast	Geo-Ko	78°57.485″N 11°31.651″E
8	London, Blomstrand Island	Lon	78°57.769″N 12°04.871″E
9	Gorilla, Blomstrand Island	Gor	78°58.401″N 12°11.857″E
10	past Zeppelin 1	Zepp1	78°55.099″N 11°57.865″E
11	past Zeppelin 2	nZepp2	78°54.933″N 11°58.780″E
13	Zeppelin tower	Zepp	78°55.280″N 11°56.872″E
14	beneath outback plateau	Hintpl	78°54.434″N 12°00.156″E
15	station Ny-Ålesund	NA	78°55.399″N 11°55.475″E
18	Björndalen	BD	78°13.167″N 15°18.777″E
20	Eindalen	Ei	78°10.784″N 15°43.099″E
21	Eindalen entrance	EiE	78°11.180″N 15°45.662″E
22	Mountain, observatory, pit 7	Berg	78°08.910″N 16°02.889″E
23	Tordalen	TD	78°10.433″N 15°53.413″E
24	Adventsdalen	AD	78°10.205″N 16°01.336″E
25	Adventsdalen, camp	ADC	78°10.292″N 16°00.574″E

*Station numbers 1–15, sampling area around Ny-Alesund; Station numbers 18–25, sampling area around Longyearbyen. For comparison see also the map of Figure 1*.

Svalbard's bedrock consists mainly of carbonate rock (Tedrow, 1977) with sandy-textured surface soil in the upper 5 cm (A-horizon). The values of soil pH range from slightly acidic to alkaline and the electrical conductivity was generally low with <100 μS cm^−1^ (Mann et al., 1986).

### BSC algal isolation and culture conditions

Samples were taken using the lower part of a Petri dish with a diameter of 6 cm, which was manually pushed about 1.5 cm into the BSC. Using a spatula the Petri dish with the sample inside was removed from the remaining BSC, closed with the lid and sealed with a stripe of Parafilm. From these BSC samples, enrichment cultures, subcultures and later algal isolates were all established on solid 1.5% Difco™ Agar (Becton Dickonson GmbH, Heidelberg, Germany) made with Bold's Basal Medium and vitamins modified by tripled nitrate concentration (3N-BBM+V) (Starr and Zeikus, 1993). All cultures were kept at 15°C, 30 μmol photons m^−2^ s^−1^ under a 16:8 h light:dark cycle (Osram Daylight Lumilux Cool White lamps L36W/840) because these parameters guaranteed suitable growth conditions which was ascertained in previous investigations. This can be explained by the assumption that most marine and terrestrial algae in the Arctic are rather migrated from the North Atlantic and hence, have relatively high temperature requirements (10–20°C) for growth and photosynthesis. In addition, a study on vegetation mats of Svalbard revealed an increase by about 5°C during summertime within the communities (Coulson et al., 1993) which shows that the culture conditions were comparable to the environmental conditions. The Agar plates were regularly inspected (twice a week) for the appearance of vital non-diatom eukaryotic algae, and positive colonies were transferred with a metal needle to a new agar plate using a stereo microscope (ZS40, Olympus, Tokyo, Japan) with a magnification of 400x. The growth of the colonies was frequently monitored and several subcultures were generated by further serial transfers under sterile conditions for purification, until no contamination with other algae or fungi was verified and unialgal cultures could be established. Using this time-consuming approach, finally 74 unialgal strains were isolated and are now kept in the Culture Collection at the University of Rostock. The isolated strains were identified to the species or at least the genus level using a light microscope (BX51, Olympus, Tokyo, Japan) with a magnification of 1,000x. The identification was mainly based on the identification key of Ettl and Gärtner (2014), and species as well as generic names were checked with Guiry and Guiry (2016)^2^ with regard to the current taxonomy. In addition, further identification literature is mentioned in Table S1. Mucilage as one identification feature was visualized with one drop of an aqueous solution of methylene blue (1.5%), and light micrographs were taken with an Olympus UC30 camera attached to the BX51 microscope and processed with the software cellSens Entry (Olympus, Tokyo, Japan). Moreover, hand drawings were made on morphological features for some algal species (Table S1).

### β-diversity

According to Whittaker (1960) β-diversity reflects the ratio between regional and local species diversity, and hence is a measure of change in species composition between habitats or variations of environmental conditions, such as moisture gradients or temperature. For this purpose, the species number was compared individually for each habitat. β-diversity is high if the species number common to both habitats is low and vice versa. Consequently, β-diversity maximum is reached if no single species common to both habitats exists, and β-diversity is at minimum if species composition of both habitats is identical. The following formula (Whittaker, 1972) was used for ß-diversity calculation using the presence-absence data of species:

$$\begin{array}{l} \beta =\left({\text{S}}_{1}-\text{c}\right)+\left({\text{S}}_{2}-\text{c}\right) \end{array}$$

S_1_ is the total number of species recorded in the first habitat, S_2_ is the total number of species recorded in the second habitat and c is thew number of species common to both communities.

### Jaccard index and Sørensen index

Both the Jaccard index (Jaccard, 1902) and Sørensen index (Sørensen, 1948) are a similarity coefficient, which measures the similarity of species composition sets. In order to calculate the Jaccard index the size of the intersection is divided by the size of the union of the sample set. The indices scales are defined from 0 to 1. The similarity is higher if the value is closer to 1. The Jaccard index (SI_J_) and the Sørensen index (SI_S_) were calculated with the following formulas:

$$\begin{array}{l} {\text{SI}}_{\text{J}}=\text{a }{\left(\text{a }+\text{ b }+\text{ c}\right)}^{-1}\\ {\text{SI}}_{\text{S}}=2\text{a }{\left(2\text{a }+\text{ b }+\text{ c}\right)}^{-1} \end{array}$$

a is the number of species common to both communities, b is the total number of species recorded in the first habitat and c is the total number of species recorded in the second habitat.

### Analyses of soil properties

The pedological variables were analyzed using the soil underneath the sampled BSCs. Determination of soil pH was performed electrometrically using a glass electrode in 0.01 M CaCl_2_ with a soil: solution ratio of 1:2.5. Total carbon (C), total nitrogen (N), and total sulphur (S) contents of the soils were determined with a Vario EL elemental analyzer (Elementar Analysensysteme GmbH, Hanau, Germany). The total phosphorus (P) content was extracted from 0.5 g dry soil material by microwave-assisted digestion with aqua regia solution (3:1 hydrochloric acid—nitric acid) (Chen and Ma, 2001). Water-extractable P was determined at a solid:solution ratio of 1:50 for 1 h at 20°C. The P concentrations in both extracts (aqua regia and water) were measured by inductively coupled plasma optical emission spectroscopy (ICP-OES) (Optima 8300 PerkinElmer LAS GmbH, Rodgau, Germany).

### Multivariate statistics

The multivariate analysis of the BSC data were conducted using the statistical programs PRIMER 6 and 7. Non-metric multi-dimensional scaling (MDS, Kruskal and Wish, 1978) was based on square root transformed data and Bray-Curtis similarity and the significance of similarity was tested by using ANOSIM permutation test (Clarke and Green, 1988; Clarke, 1993). This test calculates a global measure R which ranges between 0 and 1 and constitutes some degree of discrimination between treatments. *R* = 0 means no differences between the BSC sampling stations based on the respective species composition. *R* = 1 means that the sampling stations differ from each other. The stress value represents the quality of the graph (0 = perfect, 0.05 = good, 0.2 = poor). The MDS plot is dimensionless and visualizes the relationship of each data point to another. Distances between points represent the similarity and the difference, respectively, of all identified taxa. Principle component analysis (PCA, Kent and Coker, 1992) was based on square root transformed, normalized data and Euclidean distance matrix, and visualized comparison of the two sampling localities by chemical soil properties and precipitation data. BEST test showed environmental factor which correlated with species composition. MARGINAL test as well as SEQUETIAL test resulted from distance-based linear models (DistLM) were done to test relationships between BSC species composition and each soil parameter.

## Results

### Species composition, diversity and localities

**Figure 2:**
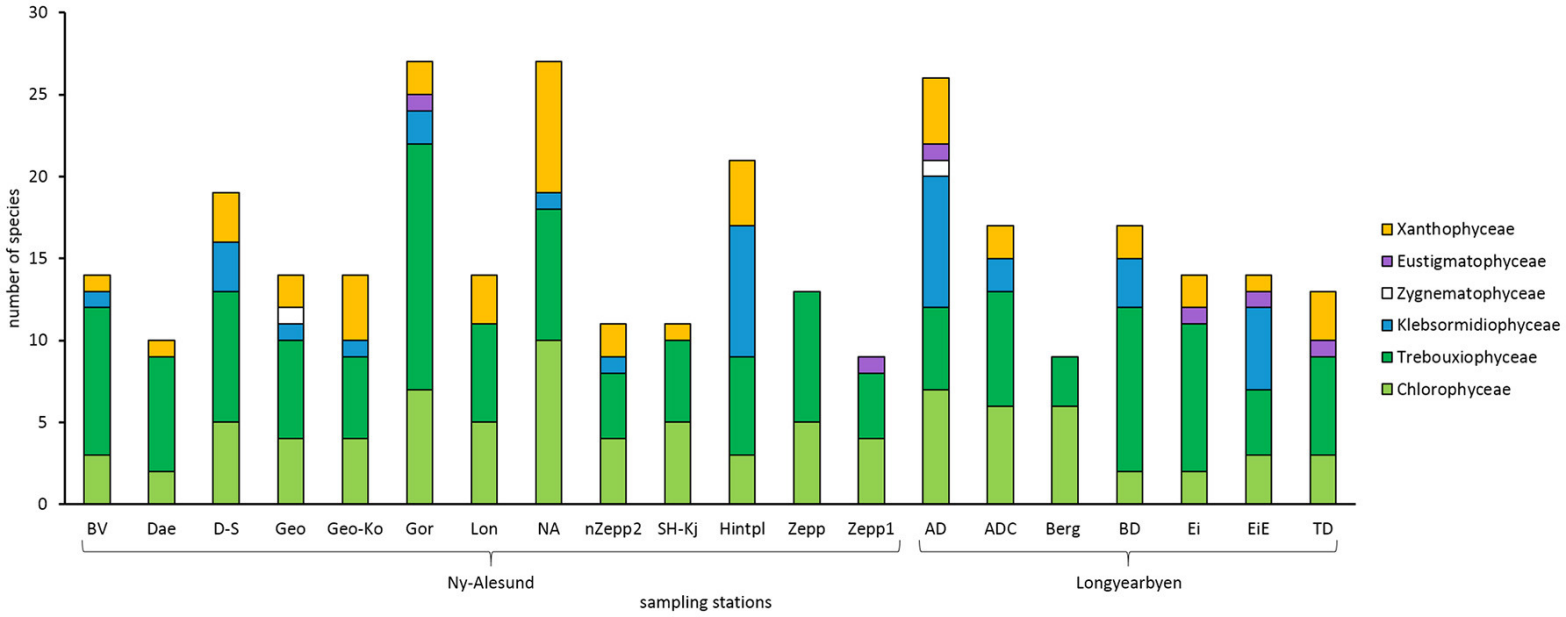
Total species number of green algae (Chlorophyceae, Trebouxiophyceae, Klebsormidiophyceae, Zygenematophyceae), Eustigmatophyceae and Xanthophyceae in all sampled biological soil crust communities on Arctic Svalbard. Abbreviations of the sampling stations refer to those in Table 1.

In total, 102 algal species were identified in the BSCs of Arctic Svalbard, with 67 species belonging to the Chlorophyta, of which 31 species were Chlorophyceae, and 36 species Trebouxiophyceae. Thirteen species were identified as Streptophyta, with 11 members of the Klebsormidiophyceae and 2 of the Zygnematophyceae, while 22 species were determined as Ochrophyta, 2 Eustigmatophyceae, and 20 Xantophyceae (Figure 2, Table 2). Species names, light micrographs, hand drawings and further information are summarized in an algae catalog (Table S1).

**Table 2:** Species list of all identified algae associated with sampled biological soil crusts on Arctic Svalbard.

**Species**	**“Ny-Alesund”**	**“Longyearbyen”**
**CHLOROPHYCEAE**		
*Bracteacoccus aggregatus*	x	
*Bracteacoccus* sp.	x	
*Chlamydomonas* sp.	x	x
*Chlorococcum lobatum*		x
*Chlorococcum* cf. *minutum*	x	
*Chlorococcum* sp.	x	
*Chloromonas* sp.	x	x
*Chromochloris zofingiensis*	x	
*Coelastrella aeroterrestrica*	x	x
*Coelastrella rubescens*	x	x
*Coelastrella* cf. *rubescens*	x	x
*Coelastrella* sp.		x
*Coenobotrys* cf. *gloeobotrydiformis*		x
*Coenochloris* sp.	x	
*Coenocystis oleifera* var. *antarctica*	x	x
*Desmodesmus abundans*	x	x
*Fasciculochloris* cf. *boldii*	x	
*Gloeocystis* sp.	x	x
*Gungnir mantoniae*	x	
*Heterotetracystis akinetos*		x
*Heterotetracystis intermedia*	x	
*Hormotilopsis* sp.		x
*Lobochlamys* cf. *culleus*	x	x
*Lobochlamys* sp.		x
*Macrochloris cohaerens*	x	
*Mychonastes homosphaera*	x	x
*Pseudodictyochloris multinucleata*	x	x
*Sporotetras polydermatica*	x	x
*Tetracystis* cf. *fissurata*	x	
Tetracystis sp.	x	x
*Uvulifera* sp.	x	
**TREBOUXIOPHYCEAE**		
*Chlorella chlorelloides*		x
*Chlorella vulgaris*	x	x
*Chlorella* cf. *vulgaris*	x	x
*Chlorella* sp.	x	x
*Chloroidium ellipsoideum*	x	x
*Chloroidium* sp.	x	
*Coccomyxa simplex*	x	x
*Coccomyxa subglobosa*	x	
*Coccomyxa* sp.	x	x
*Desmococcus olivaceus*	x	x
*Desmococcus* sp.	x	
*Dictyosphaerium dichotomum*		x
*Elliptochloris bilobata*	x	x
*Elliptochloris subsphaerica*	x	
*Elliptochloris* sp.	x	
*Gloeotila scopulina*	x	
*Heterochlorella luteoviridis*	x	
*Koliella sempervirens*	x	
*Muriella terrestris*	x	
*Muriella* sp.	x	
*Muriella* sp. I (Broady)	x	
*Myrmecia bisecta*	x	
*Myrmecia* sp.	x	
*Neocytis* cf. *brevis*	x	x
*Neocystis* sp.	x	
*Parachlorella* cf. *kessleri*		x
*Pseudochlorella* cf. *signiensis*		x
*Stichococcus allas*	x	x
*Stichococcus* cf. *allas*	x	x
*Stichococcus bacillaris*	x	x
*Stichococcus chlorelloides*	x	x
*Stichococcus exiguus*	x	
*Stichococcus* cf. *exiguus*	x	
*Stichococcus minutus*	x	x
*Stichococcus* cf. *minutus*	x	
*Stichococcus* sp.	x	x
**KLEBSORMIDIOPHYCEAE**		
*Interfilum* cf. *massjukiae*		x
*Interfilum* sp.	x	x
*Klebsormidium* cf. *crenulatum*	x	
*Klebsormidium* cf. *dissectum*	x	x
*Klebsormidium* cf. *flaccidum*	x	x
*Klebsormidium cf. klebsii*	x	
*Klebsormidium* cf. *montanum*	x	x
*Klebsormidium* cf. *nitens*	x	x
*Klebsormidium* cf. *subtile*		x
*Klebsormidium* sp.	x	x
*Klebsormidium* sp. (G-Clade)	x	x
**ZYGNEMATOPHYCEAE**		
*Actinotaenium* sp.		x
*Cylindrocystis crassa*	x	
Eustigmatophyceae		
*Eustigmatos vischeri*	x	x
*Eustigmatos* cf. *vischeri*		x
**XANTHOPHYCEAE**		
*Botrydiopsis intercedens*	x	
*Botrydiopsis arhiza*	x	
*Botrydiopsis* cf. *constricta*	x	
*Chlorellidium* cf. *tetrabotrys*		x
*Chloridella sp*.	x	
*Heterococcus* sp.	x	x
*Monallantus brevicylindrus*	x	
*Nephrodiella* cf. *phaseolus*	x	
*Pleurochloris meiringensis*	x	x
*Pleurochloris pseudopolychloris*	x	x
*Pleurochloris polychloris*	x	
*Pleurogaster lunaris*	x	x
*Tribonema viride*	x	
*Tribonema vulgare*	x	
*Tribonema* sp.	x	
*Xanthonema* cf. *debile*	x	x
*Xanthonema solidum*	x	x
*Xanthonema* cf. *solidum*	x	
*Xanthonema exile*	x	
*Xanthonema* sp.	x	x

*Information about the two sampling areas refers to those in Table 1. x: Presence of the respective algae species*.

BSC algal species numbers varied between 9 and 27 taxa per sampling station (Figure 2). At most sampling stations species number ranged between 9 and 14 taxa. Species richness of five sampling stations around Ny-Ålesund (D-S, Gor, NA, Hinter, AD) was about double as high.

In addition, the proportion of taxonomic groups was also variable. All investigated BSCs contained Chlorophyceae as well as Trebouxiophyceae while Zygenematophyceae were confined to only 2 sampling stations and Eustigmatophyceae to 3 stations. Moreover, Xanthophyceae were determined in all BSCs except 3 sampling stations. Interestingly, BSCs from “Zepp” and “Berg” hosted exclusively Chlorophyceae and Trebouxiophyceae.

However, the composition differed and 3 common taxa were found. The most abundant algal species, which were found in at least 10 sampling stations, were *Coccomyxa simplex* Mainx, *Coccomyxa* sp., *Mychonastes homosphaera* Fott and Novákova, and *Stichococcus bacillaris* Grintzesco and Péterfi. It should be pointed out that none of the more common species could be identified in all sampled BSCs.

Surprisingly, *Klebsormidium* strains belonging to clade G (Streptophyta) were for the first time determined in the Arctic at 5 sampling stations which were mainly located around Longyearbyen (Figure 2, Table S1). Species of *Klebsormidium* usually are very difficult to distinguish by morphology and hence genetic analysis are necessary. However, members of clade G exhibit unique morphological features (Rindi et al., 2011), for example, they form thin filaments (4.5–8 μm wide) with short but compact cells and small pyrenoid.

For the Ny-Ålesund and Longyearbyen data set a β-diversity value of 64 was calculated and the Jaccard index as well as Sørensen index were lower than 0.4 (Table 3) pointing to differences in species numbers between both sampling areas which was additionally visualized by a frequency histogram (Figure S1).

**Table 3:** Diversity indices.

	**“Ny-Alesund”–“Longyearbyen”**
β-diversity	64
Jaccard index	0.22
Sørensen index	0.36

*The scales of Jaccard index and Sørensen index are defined from 0 to 1 (0 = no similarity, 1 = highest similarity)*.

**Figure 3:**
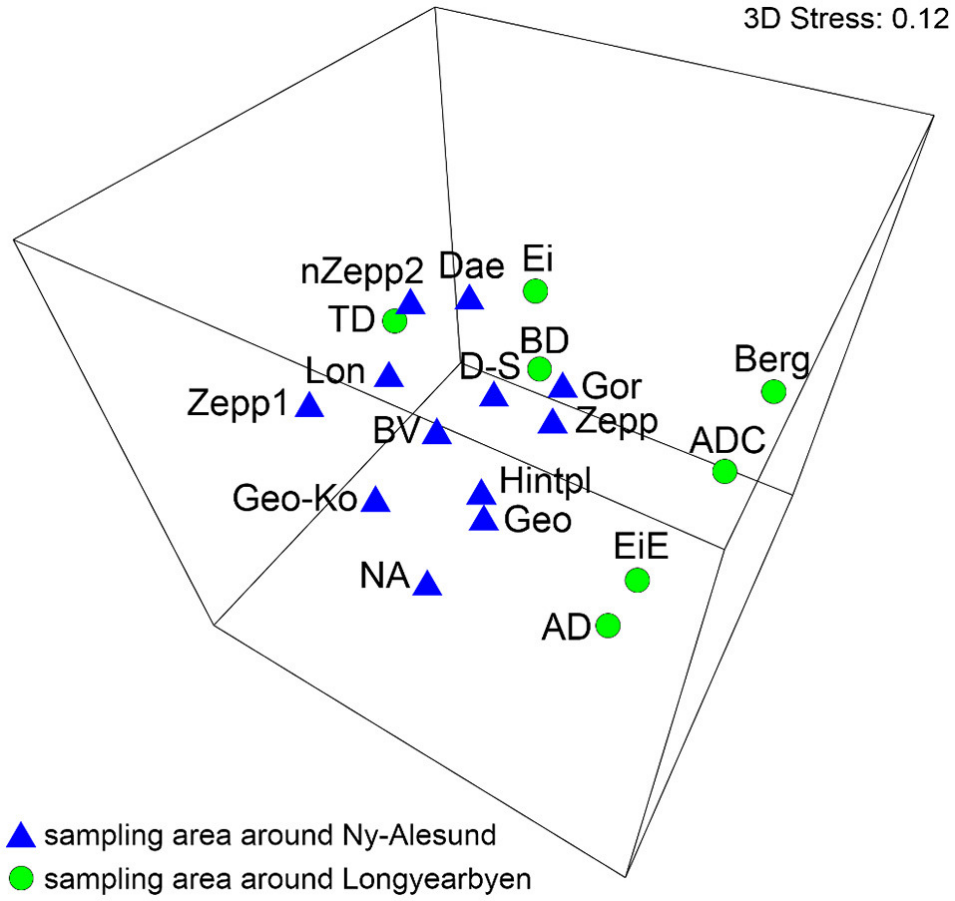
MDS plot based on square root transformed data and Bray-Curtis similarity. Comparison of the two sampling localities by their species. The stress value represents the quality of the graph (0 = perfect, 0.05 = good, 0.2 = poor). Abbreviations of the sampling stations refer to those in Table 1.

The MDS analysis showed significantly no grouping by sampling localities regarding the species compositions (Figure 3). ANOSIM test resulted in global *R*-value of 0.267 with a significance of *p* = 0.008 which confirmed that significantly no differentiation between the BSC species composition from sampling stations around Ny-Ålesund and Longyearbyen existed.

### Soil properties

The pH value ranged from extremely acidic (4.2) to slightly alkaline (7.4), with predominance of neutral to slightly alkaline in the tested areas. The soil organic matter content ranged from the level of mineral soils in BSCs from “NA” (calculated soil organic matter content: <3%) to the level of peaty soils (>50% soil organic matter) in “BV” (calculated soil organic matter content: 70%). BSCs from “Zepp1” had the highest S content (about 1%) whereby the median value of the S content was 0.1%. The TP ranged between low contents of 112.8 mg kg^−1^ in samples from “Geo” and high contents of 782.8 mg kg^−1^ in samples from “BV.” In “BV” nearly half of the TP (381.3 mg kg^−1^) was water-extractable and thereby easily plant-available (Table 4).

**Table 4:** Chemical properties of sampled soil underneath biological soil crusts on Arctic Svalbard.

**Sampling station**	**Precipitation [mm a^−1^]^a^**	**pH value**	**C [g kg^−1^]**	**N [g kg^−1^]**	**S [g kg^−1^]**	**TP [mg kg^−1^]**	**Water-extractable P [mg kg^−1^]**
AD	205	4.9	98.8	6.6	2.5	581.3	20.4
ADC	205	7.0	38.2	2.5	0.7	753.0	87.3
Berg	205	6.1	26.0	1.9	0.8	461.9	4.5
BD	471	5.9	111.6	3.6	0.6	543.2	48.6
BV	205	4.7	403.6	19.9	3.0	782.8	381.3
Dae	471	4.9	20.7	1.5	0.6	365.7	10.6
D-S	471	6.9	108.5	3.4	0.6	334.2	8.4
Ei	205	4.2	74.9	2.9	2.9	281.6	0.1
EiE	205	6.8	331.6	9.6	4.9	395.0	4.4
Geo	471	7.2	88.6	1.5	0.6	112.8	2.4
Geo-Ko	471	7.4	101.4	3.6	1.7	155.3	7.7
Gor	471	5.0	90.5	5.0	1.4	315.3	17.7
Lon	471	7.2	104.7	3.2	0.6	359.8	24.8
NA	471	6.3	17.0	1.4	0.5	169.1	5.5
NA without BSCs	471	6.5	101.4	3.6	1.7	173.4	7.0
nZepp2	471	6.0	27.2	1.8	1.2	286.5	12.1
SH-Kj	471	7.2	183.1	11.0	3.3	1418.7	21.2
TD	205	5.5	296.2	12.1	6.4	515.9	108.1
Hintpl	471	7.2	109.4	5.1	1.4	522.1	58.6
Zepp	471	5.2	385.4	14.3	4.5	438.5	174.4
Zepp1	471	6.2	346.9	10.6	10.8	101.1	12.6

*Precipitation data for the sampling regions are also given. Abbreviations of the sampling stations refer to those in Table 1*.

a *Information from Norwegian Meteorological Institute*.

*C, carbon; N, nitrogen; S, sulfur; TP, total Phosphorus*.

Additionally, the soil properties of BSCs-free topsoil in “NA” were analyzed in order to compare bare soil and sediment underneath BSCs and thus, to investigate influences of these communities. These BSCs-free soil was characterized as reference by soil organic matter accumulation already (calculated soil organic matter content about 17 vs. 3% in the BSC covered soil) and an increased P content (Table 4).

### Species composition and soil properties

**Figure 4:**
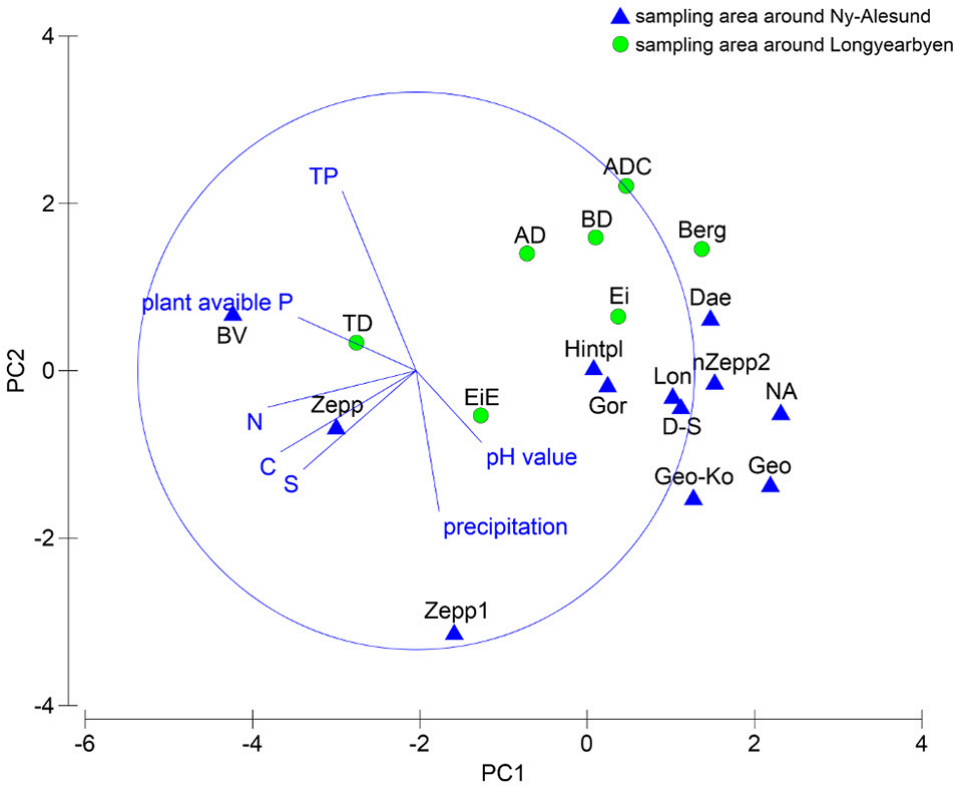
PCA plot based on square root transformed, normalized data and Euclidean distance matrix. Comparison of the two sampling localities by chemical soil properties and precipitation data.

The relationship between species composition of each sampling station and the respective soil properties were analyzed statistically. PCA showed no clear clusters (Figure 4) and ANOSIM permutation test calculated a global *R*-value of 0.299 and a *p*-value of 0.019. The direction of the vectors in the plot visualized at which sampling station the parameter had a greater influence. The BEST test ascertained the environmental parameter which had an influence on the species composition and revealed correlation with precipitation. Both MARGINAL test and SEQUETIAL test confirmed this result by a significant *p*-value of 0.011. No further correlations with the other analyzed factors could be determined but a *p*-value of 0.061 indicated that a relationship between pH value and species composition might exist.

### Comparison between arctic and antarctic species composition and diversity

**Figure 5:**
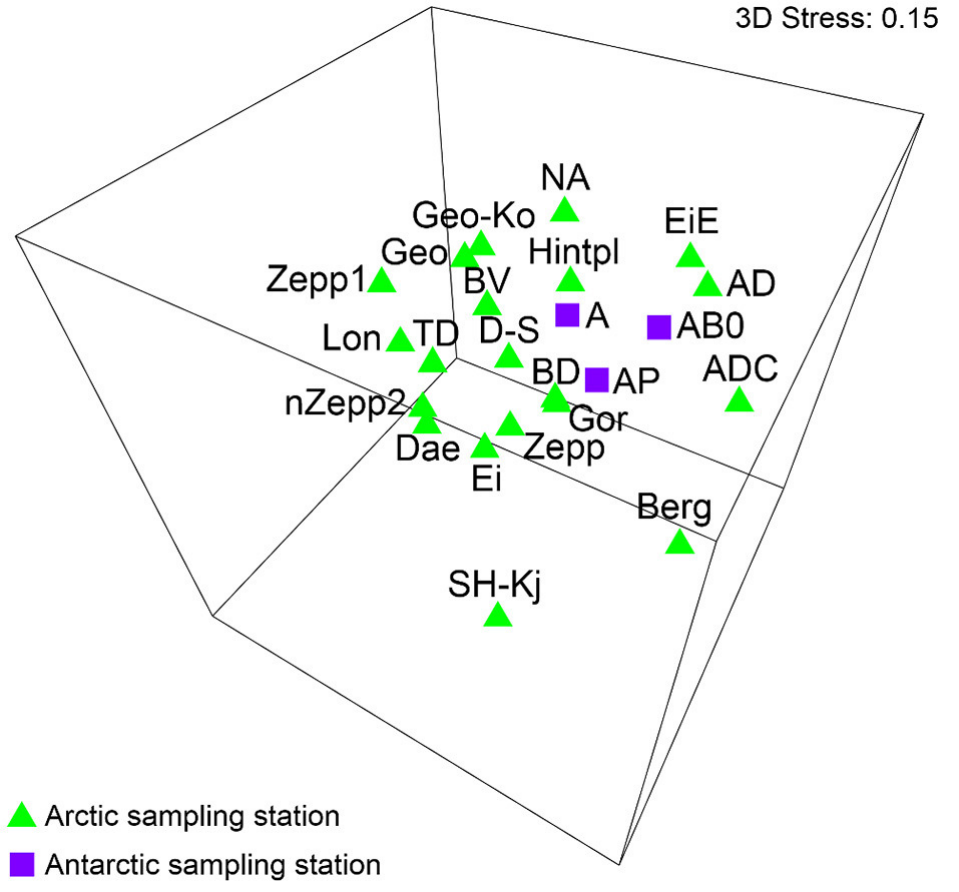
MDS plot based on square root transformed data and Bray-Curtis similarity. Comparison of the Arctic and Antarctica by their species. The stress value represents the quality of the graph (0 = perfect, 0.05 = good, 0.2 = poor). Abbreviations of the sampling stations refer to those in Table 1.

Data sets of Antarctic BSC algal species reported by Borchhardt et al. (2017) were used for statistical analyses in order to compare species composition of both Polar Regions. The authors identified 106 taxa in maritime Antarctica and calculated β-diversity value of 61–67. These results showed very similar algal species richness and diversity of BSC to data presented in the present study. A comparison of both species lists revealed 40 common taxa in overall 112 species. The MDS analysis including datasets of Arctic as well as Antarctic BSCs visualized no significant grouping by Polar Regions (Figure 5). ANOSIM test calculated global *R*-value of 0.092 which indicated that no differentiation between the BSC species composition from Svalbard and maritime Antarctica existed. However, lack of significance could be confirmed because the significance level of sample statistic was *p* = 0.283.

## Discussion

BSCs host a variety of different organisms (reviewed in Belnap et al., 2003; Weber et al., 2016). Compared to former literature, the data presented in this study point to a surprisingly high species richness of microalgae in BSCs of Arctic Svalbard (102 species). Studies on terrestrial microflora are rare in comparison to aquatic investigations (Elster et al., 1999) and also BSCs are generally poorly studied in the Polar Regions (Green and Broady, 2001). Previous studies reported different species numbers of microalgae in BSCs which ranged from only few to about 100 taxa depending on habitat. A study about the algal species composition of continental Antarctica reported only 10 chlorophytes in soil samples (Broady and Weinstein, 1998) although they were not associated with a BSC community. In contrast, 78 eukaryotic algal species in soil (Zidarova, 2008) and 106 species in BSCs (Borchhardt et al., 2017) of maritime Antarctica were reported. Kim et al. (2008) identified 23 eukaryotic algal species and (Kaštovská et al., 2005) 33 taxa in soil samples from Arctic Svalbard partly covered by vascular plants and mosses which point to a rather low diversity. In addition, Elster et al. (1999) found 84 eukaryotic soil algae in the Arctic desert of central Ellesmere Island, Canada which is similar to the data presented in this study. However, a comparison of both species lists revealed less matches, only 33 taxa (29 green algae, 4 xanthophytes) were determined in both study sites.

The proportion of xanthophyte species was 5 fold higher (20 species) in the present study compared to the reported number in BSCs from coastal dunes at the Baltic Sea (Schulz et al., 2016, 4 species). This conspicuous biogeographic pattern for xanthophytes was already indicated by Büdel et al. (2016), who assumed that the species richness of xanthophytes increases with cooler habitats. These authors mentioned members of *Botrydiopsis, Tribonema, Xanthonema*, and *Heterococcus* as most common species which all occurred in the Arctic BSCs of the present study. Lower species numbers of xanthophytes were determined in the Arctic desert of Canada (4 species, Elster et al., 1999) and Maritime Antarctica (7 species, Borchhardt et al., 2017).

Unfortunately, some aspects are impeding detailed comparison with previous studies. On one hand more investigations on the biodiversity of BSCs at high latitudes are needed and on the other hand sometimes only species numbers without taxonomic information were provided and thus verification was not possible. Furthermore, identification by morphology is often difficult and depends on experience, methods, equipment and taxonomic state of the organisms, or cryptic species. Therefore, we suggest to use an integrative approach including several methods such as direct microscopy and cultivation with different media and additionally genetic analyses. Metagenomic approaches for Arctic BSCs were not conducted yet, because of still unsolved problems with optimum primers and available sequences in database. On the other hand comprehensive data based on morphology exist in the literature which enabled a comparison with our results.

Fortunately, a direct comparison of species richness and biodiversity of Arctic as well as Antarctic BSCs could be performed because the identifications were done by the same authors using exactly the same methods. These analyses revealed approximately equal β-diversity based on very similar species numbers (Arctic: 102, Antarctic: 106) and therefore, we conclude that the species richness of BSCs in the Polar Regions are congruent. Moreover, MDS as well as ANOSIM analysis showed no significant grouping by Polar Regions which indicated that many BSC taxa (40 species) are bipolar distributed. Some of these bipolar taxa are also known as cosmopolitans, for example, member of *Chlamydomonas, Coccomyxa*, and *Klebsormidium* (Ettl and Gärtner, 2014).

Surprisingly, *Klebsormidium* sp. (Streptophyta) belonging to G clade (according to Rindi et al., 2011) was found at 5 sampling stations on Arctic Svalbard. In general, the genus *Klebsormidium* is distributed worldwide as a typical member of BSC communities, and exhibits a high level of morphological plasticity which is shown as differences in ultrastructure and cell wall texture (Mikhailyuk et al., 2014). Due to this peculiarity, the delimitation of species by morphology usually is very difficult and therefore, genetic analysis are necessary. In contrast, the G lineage is genetically relatively isolated from all other *Klebsormidium* species and additionally, also distinguished from the other lineages by a unique morphology. Therefore, it is possible to unambiguously identify members of G clade using light microscopy only. Most interesting, species belonging to G clade were so far mainly found in BSCs of hot dryland regions of South Africa (Rindi et al., 2011; Karsten et al., 2015; Mikhailyuk et al., 2015). All these authors characterized members of clade G as African group and Ryšánek et al. (2015) confirmed that an investigation of 200 *Klebsormidium* strains from Europe, Asia and North America revealed no single species belonging to clade G. South Africa, where the strains of clade G were isolated for the first time, constitutes a xerophytic habitat which is characterized by very low annual precipitation of 56 mm with only 0–14 monthly rainfall days and hence by the occurrence of desiccation-tolerant microalgae such as *Klebsormidium* clade G members (Karsten et al., 2015). Statistical analysis of the biogeography of several *Klebsormidium* species from different regions showed clear separation of the African from the remaining groups and hence very high dissimilarity (Mikhailyuk et al., 2015). For the first time in the present study 5 clade G *Klebsormidium* strains could be identified in samples mainly collected around Longyearbyen which has also low annual precipitation (205 mm), and hence can be characterized as dry Tundra climate ET according the climate classification by Köppen and Geiger (1930–1939). A study on the biodiversity of *Klebsormidium* taxa along elevational gradients of the Alps demonstrated that members of all known lineages (B, C, D, E, and F) could be morphologically and genetically verified except members of clade G (Mikhailyuk et al., 2015). Data of this investigation indicated that the key driver for the occurrence of *Klebsormidium* clade G is rather water availability than the temperature which coincides with our results.

Although the species composition of all sampled BSCs from Svalbard highly varied, our results showed no significant differences between the two investigated localities Ny-Alesund and Longyearbyen. Therefore the biodiversity data might be representative for a larger area of Arctic Svalbard.

No correlations between BSC species composition and analyzed chemical soil properties could be outlined, although Schulz et al. (2016) reported the TP content as the main driver for the BSC microalgal species composition collected at coastal dunes of the Baltic Sea. This might be explained by generally higher P contents and dominant optimal soil pH for high bioavailability of P (Table 4) in these Arctic soils, which suggest a sufficient P supply instead of P limited growth. A comparison with the soil TP concentrations measured by Schulz et al. (2016) showed noticeable lower values, with TP contents ranging between 90 and 310 mg kg^−1^ soil with a median value of 110 mg kg^−1^. In contrast, our analysis resulted in about 3 fold higher TP contents (median of 366 mg kg^−1^) with a remarkable maximum value of 753 mg kg^−1^. Mann et al. (1986) analyzed soils from different locations around Kongsfjorden which corresponded to the sampling stations “Geo” and “London” located near Ny-Ålesund. The TP contents of the soil directly underneath the BSCs in the present study revealed about 10-fold increased P amounts compared to the bulk topsoil data presented by Mann et al. (1986). Our TP concentrations are confirmed by results of Beraldi-Campesi et al. (2009), who also described increased P contents under BSCs of the Colorado Plateau highlands and the Sonoran Desert lowlands (USA) compared to the bulk soil without BSCs, as well as by increased P concentrations with BSC growth (Wu et al., 2013). Chemical data of soil underneath BSCs presented in this study revealed noticeable higher contents of organic matter, which might be promoted by decreased decomposition activity of soil organic matter in Arctic climate conditions, which is confirmed by observations in Arctic and Antarctic BSCs (Williams et al., 2016).

The determined pH values varied between 4.2 and 7.4 but significant correlation with BSC algal species composition was not confirmed statistically. However, Kaštovská et al. (2007) reported that the abundance of microalgae increased with lower pH, and data of statistical analysis presented in our study showed a *p*-value very close to significance threshold which might indeed reflect such a relationship between pH and species composition. It should be pointed out that our results were based on presence-absence data, and that pH might affect more the abundance of organisms than species richness.

Only precipitation could be identified as one key factor which controlled BSC species composition and it is known that the availability of water is an important environmental factor for the occurrence of soil algae (Hoffmann, 1989; Ohtani et al., 1991; reviewed in Broady, 1996; Adams et al., 2006). Borchhardt et al. (2017) also showed that soil moisture content of various Antarctic microhabitats affected the specific species composition in BSCs.

Another abiotic factor, which controls BSC composition, might be substrate and soil texture. Results by Kaštovská et al. (2005) and Schulz et al. (2016) indicated that the soil particle size influenced the abundance as well as diversity of BSC organisms.

The discrepancy of missing correlation between species compositions and environmental factors (except water availability) can be explained by the heterogeneity of the studied habitats. Both microclimatic (e.g., diurnal variations) and macroclimatic factors (e.g., precipitation and temperature) might affect species diversity. However, more studies are needed to evaluate which environmental factors control species composition and biodiversity in Arctic BSCs. These results in combination with the provided species lists would offer a basis for understanding the functions of BSCs which constitute the main vegetation of Arctic Svalbard.

## Author contributions

UK conceived and designed the study. UK and NB collected the samples. NB cultivated as well as isolated all microalgae, took light micrographs, made hand drawings, created figures and tables, calculated biodiversity indices, did statistical analyses and wrote the first draft of the manuscript. NB and TM identified species. CB did all soil analyses and wrote part of the section Material and Methods. All authors edited and revised the manuscript and approved the publication.

### Conflict of interest statement

The authors declare that the research was conducted in the absence of any commercial or financial relationships that could be construed as a potential conflict of interest.

## Footnotes

^1^https://www.met.no

^2^http://algaebase.org

## Supplementary material

Table S1 Algae catalog. Species list of algae and summary of several information about every identified species. x: Presence of the species, black arrow: nucleus, white arrow: pyrenoid, gray arrow: stigma, bar: 10 μm.

Figure S1 Frequency histogram. Number of species which were found in different numbers of sampling stations on Svalbard.

## References

[B1] Adams B. J. Bardgett R. D. Ayres E. Wall D. H. Aislabie J. Bamforth S. (2006). Diversity and distribution of Victoria Land biota. Soil Biol. Biochem. 38, 3003–3018. 10.1016/j.soilbio.2006.04.030

[B2] Barberousse H. Ruiz G. Gloaguen V. Lombardo R. J. Djediat C. Mascarell G.. (2006). Capsular polysaccharides secreted by building façade colonisers: characterisation and adsorption to surfaces. Biofouling 22, 361–370. 10.1080/08927010601035803 17178569

[B3] Baumann K. Glaser K. Mutz J.-E. Karsten U. MacLennan A. Hu Y. (2017). Biological soil crusts of temperate forests: their role in P cycling. Soil Biol. Biochem. 109, 156–166. 10.1016/j.soilbio.2017.02.011

[B4] Belnap J. (2002). Nitrogen fixation in biological soil crusts from southeast Utah, U. S. A. Biol. Fertil. Soils 35, 128–135. 10.1007/s00374-002-0452-x

[B5] Belnap J. (2003). Factors influencing nitrogen fixation and nitrogen release in biological soil crusts, in Biological Soil Crusts: Structure, Function, and Management, eds Belnap J. Lange O. L. (Berlin; Heidelberg: Springer), 241–261.

[B6] Belnap J. (2006). The potential roles of biological soil crusts in dryland hydrologic cycles. Hydrol. Process. 20, 3159–3178. 10.1002/hyp.6325

[B7] Belnap J. Gillette D. A. (1998). Vulnerability of desert biological soil crusts to wind erosion: the influences of crust development, soil texture, and disturbance. J. Arid Environ. 39, 133–142. 10.1006/jare.1998.0388

[B9] Belnap J. Büdel B. Lange O. L. (2001). Biological Soil Crusts: Characteristics and Distribution. Berlin; Heidelberg: Springer.

[B8] Belnap J. Lange O. (2003). Biological Soil Crusts: Structure, Function, and Management. Berlin; Heidelberg: Springer.

[B10] Belnap J. Prasse R. Harper K. T. (2003). Influence of biological soil crusts on soil environments and vascular plants, in Biological Soil Crusts: Structure, Function, and Management, eds Belnap J. Lange O. L., (Berlin; Heidelberg: Springer), 281–300.

[B11] Beraldi-Campesi H. Hartnett H. E. Anbar A. Gordon G. W. Garcia-Pichel F. (2009). Effect of biological soil crusts on soil elemental concentrations: implications for biogeochemistry and as traceable biosignatures of ancient life on land. Geobiology 7, 348–359. 10.1111/j.1472-4669.2009.00204.x 19573165

[B12] Bhatnagar A. Makandar M. B. Garg M. K. Bhatnagar M. (2008). Community structure and diversity of cyanobacteria and green algae in the soils of Thar Desert (India). J. Arid Environ. 72, 73–83. 10.1016/j.jaridenv.2007.05.007

[B13] Bock C. Krienitz L. Pröschold T. (2011). Taxonomic reassessment of the genus Chlorella (Trebouxiophyceae) using molecular signatures (barcodes), including description of seven new species. Fottea 2, 293–312. 10.5507/fot.2011.028

[B14] Borchhardt N. Schiefelbein U. Abarca N. Boy J. Mikhailyuk T. Sipman H. J. M. (2017). Diversity of algae and lichens in biological soil crusts of Ardley and King George islands, Antarctica. Antarct. Sci. 9, 1–9. 10.1017/S0954102016000638

[B15] Breen K. Lévesque E. (2008). The influence of biological soil crusts on soil characteristics along a high Arctic Glacier Foreland, Nunavut, Canada. Arctic Antarct Alp. Res. 40, 287–297. 10.1657/1523-0430(06-098)[BREEN]2.0.CO;2

[B16] Broady P. A. (1996). Diversity, distribution and dispersal of Antarctic terrestrial algae. Biodivers. Conserv. 5, 1307–1335. 10.1007/BF00051981

[B17] Broady P. A. Weinstein R. N. (1998). Algae, lichens and fungi in La Gorce Mountains, Antarctica. Antarct. Sci. 10, 376–385. 10.1017/S0954102098000467

[B18] Büdel B. Darienko T. Deutschewitz K. Dojani S. Friedl T. Mohr K. I.. (2009). Southern African biological soil crusts are ubiquitous and highly diverse in drylands, being restricted by rainfall frequency. Microb. Ecol. 57, 229–247. 10.1007/s00248-008-9449-9 18850242

[B19] Büdel B. Dulić T. Darienko T. Rybalka N. Friedl T. (2016). Cyanobacteria and algae of biological soil crusts, in Biological Soil Crusts: An Organizing Principle in Drylands, eds Weber B. Büdel B. Belnap J. (Berlin: Springer International Publishing), 55–80.

[B20] Chen M. Ma L. Q. (2001). Comparison of three aqua regia digestion methods for twenty Florida soils. Soil Sci. Am. J. 65, 491–499. 10.2136/sssaj2001.652491x

[B21] Clarke K. R. (1993). Non-parametric multivariate analyses of changes in community structure. Aust. J. Ecol. 18, 117–143. 10.1111/j.1442-9993.1993.tb00438.x

[B22] Clarke K. R. Green R. H. (1988). Statistical design and analysis for a ‘Biological Effect’ study. Mar. Ecol. Prog. Ser. 46, 213–126. 10.3354/meps046213

[B23] Colesie C. Gommeaux M. Green T. G. A. Buedel B. (2014). Biological soil crusts in continental Antarctica: Garwood Valley, southern Victoria Land, and Diamond Hill, Darwin Mountains region. Antarct. Sci. 26, 115–123. 10.1017/S0954102013000291

[B24] Coulson S. Hodkinson I. D. Strathdee A. Bale J. S. Block W. Worland M. R. (1993). Simulated climate change: the interaction between vegetation type and microhabitat temperatures at NyÅlesund, Svalbard. Polar Biol. 13, 67–70.

[B25] Darienko T. Gustavs L. Pröschold T. (2016). Species concept and nomenclatural changes within the genera Elliptochloris and Pseudochlorella (Trebouxiophyceae) based on an integrative approach. J. Phycol. 52, 1125–1145. 10.1111/jpy.12481 27734501

[B26] Darienko T. Gustavs L. Eggert A. Wolf W. Pröschold T. (2015). Evaluating the species boundaries of green microalgae (Coccomyxa, Trebouxiophyceae, Chlorophyta) using integrative taxonomy and DNA barcoding with further implications for the species identification in environmental samples. PLoS ONE 10:e0127838. 10.1371/journal.pone.0127838 26080086 PMC4469705

[B27] Darienko T. Gustavs L. Mudimu O. Menendez C. R. Schumann R. Karsten U. (2010). Chloroidium, a common terrestrial coccoid green alga previously assigned to Chlorella (Trebouxiophyceae, Chlorophyta). Eur. J. Phycol. 45, 79–95. 10.1080/09670260903362820

[B28] Eldridge D. Greene R. (1994). Microbiotic soil crusts - a review of their roles in soil and ecological processes in the rangelands of Australia. Aust. J. Soil Res. 32:389 10.1071/SR9940389

[B29] Elster J. Lukesová A. Svoboda J. Kopecky J. Kanda H. (1999). Diversity and abundance of soil algae in the polar desert, Sverdrup Pass, central Ellesmere Island. Polar Rec. 35, 231 10.1017/S0032247400015515

[B30] Ettl H. Gärtner G. (2014). Syllabus der BODEN-, Luft- und Flechtenalgen. Berlin; Heidelberg: Springer.

[B31] Evans R. D. Belnap J. (1999). Long-term consequences of disturbance on nitrogen dynamics in an arid ecosystem. Ecology 80, 150–160. 10.1890/0012-9658(1999)080[0150:LTCODO]2.0.CO;2

[B32] Evans R. D. Lange O. L. (2001). Biological soil crusts and ecosystem nitrogen and carbon dynamics. Ecol. Stud. 150, 263–279. 10.1007/978-3-642-56475-8_20

[B33] Frenot Y. Chown S. L. Whinam J. Selkirk P. M. Convey P. Skotnicki M.. (2005). Biological invasions in the Antarctic: extent, impacts and implications. Biol. Rev. 80, 45–72. 10.1017/S1464793104006542 15727038

[B34] Fucikova K. Lewis L. A. (2012). Intersection of Chlorella, Muriella and Bracteacoccus: resurrecting the genus Chromochloris Kol et Chodat (Chlorophyceae, Chlorophyta). Fottea 12, 83–93. 10.5507/fot.2012.007

[B35] Fucikova K. Flechtner V. R. Lewis L. A. (2012). Revision of the genus Bracteacoccus Tereg (Chlorophyceae, Chlorophyta) based on a phylogenetic approach. Nov. Hedwigia 96, 15–59. 10.1127/0029-5035/2012/0067

[B36] Ghiloufi W. Büdel B. Chaieb M. (2016). Effects of biological soil crusts on a Mediterranean perennial grass (*Stipatanacissima*, L.). Plant Biosyst. 151, 158–167. 10.1080/11263504.2015.1118165

[B37] Green T. G. A. Broady P. A. (2001). Biological soil crusts of Antarctica. Ecol. Stud. 150, 133–139. 10.1007/978-3-642-56475-8_11

[B38] Guiry M. D. Guiry G. M. (2016). Algaebase. Galway: World-wide electronic publication, National University of Ireland Available online at: http://www.algaebase.org

[B39] Harper K. T. Belnap J. (2001). The influence of biological soil crusts on mineral uptake by associated vascular plants. J. Arid Environ. 47, 347–357. 10.1006/jare.2000.0713

[B40] Hoffmann L. (1989). Algae of terrestrial habitats. Bot. Rev. 55, 77–105. 10.1007/BF02858529

[B41] Huss V. A. R. Frank C. Hartmann E. C. Hirmer M. Kloboucek A. Seidel B. M. (1999). Biochemical taxonomy and molecular phylogeny of the genus Chlorella sensu lato (Chlorophyta). J. Phycol. 35, 587–598. 10.1046/j.1529-8817.1999.3530587.x

[B42] Jaccard P. (1902). Lois de distribution florale. Bull. Soc. Vaud. Sci. Nat. 38, 69–130.

[B43] Karsten U. Herburger K. Holzinger A. (2014). Dehydration, temperature, and light tolerance in members of the aeroterrestrial green algal genus Interfilum (Streptophyta) from biogeographically different temperate soils. J. Phycol. 50, 804–816. 10.1111/jpy.12210 25810561 PMC4370238

[B44] Karsten U. Herburger K. Holzinger A. (2015). Living in biological soil crust communities of African deserts – physiological traits of green algal *Klebsormidium* species (Streptophyta) to cope with desiccation, light and temperature gradients. J. Plant Physiol. 194, 2–12. 10.1016/j.jplph.2015.09.002 26422081 PMC4710676

[B45] Kaštovská K. Elster J. Stibal M. Santrucková H. (2005). Microbial assemblages in soil microbial succession after glacial retreat in Svalbard (high arctic). Microb. Ecol. 50, 396–407. 10.1007/s00248-005-0246-4 16328651

[B46] Kaštovská K. Stibal M. Šabacká M. Černá B. Šantrčková H. Elster J. (2007). Microbial community structure and ecology of subglacial sediments in two polythermal Svalbard glaciers characterized by epifluorescence microscopy and PLFA. Polar Biol. 30, 277–287. 10.1007/s00300-006-0181-y

[B47] Kaufnerová V. Eliáš M. (2013). The demise of the genus Scotiellopsis Vinatzer (Chlorophyta). Nov. Hedwigia 97, 415–428. 10.1127/0029-5035/2013/0116

[B48] Kent M. Coker P. (1992). Vegetation Description and Analysis: A Practical Approach. Chichester: John Wiley & Sons.

[B49] Kessler E. Schäfer M. Hümmer C. Kloboucek A. Huss V. A. R. (1997). Physiological, biochemical, and molecular characters for the taxonomy of the subgenera of Scenedesmus (Chlorococcales, Chlorophyta). Bot. Acta 110, 244–250. 10.1111/j.1438-8677.1997.tb00636.x

[B50] Kim G. H. Klochkova T. A. Kang S. H. (2008). Notes on freshwater and terrestrial algae from Ny-Ålesund, Svalbard (high Arctic sea area). J. Environ. Biol. 29, 485–491. 19195386

[B51] Köppen W. Geiger G. (1930–1939). Handbuch der Klimatologie. Berlin: Gebrüder Borntraeger.

[B52] Kostikov I. Darienko T. Lukešova A. Hoffmann L. (2002). Revision of the classification system of Radiococcaceae Fott ex Komárek (except the subfamily Dictyochlorelloideae) (Chlorophyta). Algol. Stud. 104, 23–58.

[B53] Krienitz L. Hegewald E. H. Hepperle D. Huss V. A. R. Rohr T. Wolf M. (2004). Phylogenetic relationship of Chlorella and Parachlorella gen. nov. (Chlorophyta, Trebouxiophyceae). Phycologia 43, 529–542. 10.2216/i0031-8884-43-5-529.1

[B54] Kruskal J. B. Wish M. (1978). Multidimensional scaling. Soc. Sci. 11, 19–34. 10.4135/9781412985130

[B55] Langhans T. M. Storm C. Schwabe A. (2009). Community assembly of biological soil crusts of different successional stages in a temperate sand ecosystem, as assessed by direct determination and enrichment techniques. Microb. Ecol. 58, 394–407. 10.1007/s00248-009-9532-x 19479305

[B56] Mann D. H. Sletten R. S. Ugolini F. C. (1986). Soil development at Kongsfjorden, Spitsbergen. Polar Res. 4, 1–16. 10.3402/polar.v4i1.6914

[B57] Mikhailyuk T. I. Sluiman H. J. Massalski A. Mudimu O. Demchenko E. M. Kondratyuk S. Y.. (2008). New streptophyte green algae from terrestrial habitats and an assessment of the genus Interfilum (Klebsormidiophyceae, Streptophyta). J. Phycol. 44, 1586–1603. 10.1111/j.1529-8817.2008.00606.x 27039871

[B58] Mikhailyuk T. Glaser K. Holzinger A. Karsten U. (2015). Biodiversity of Klebsormidium (Streptophyta) from alpine biological soil crusts (Alps, Tyrol, Austria, and Italy). J. Phycol. 51, 750–767. 10.1111/jpy.12316 26504252 PMC4618304

[B59] Mikhailyuk T. Holzinger A. Massalski A. Karsten U. (2014). Morphology and ultrastructure of Interfilum and Klebsormidium (Klebsormidiales, Streptophyta) with special reference to cell division and thallus formation. Eur. J. Phycol. 49, 395–412. 10.1080/09670262.2014.949308 26504365 PMC4618308

[B60] Molinari-Novoa E. A. (2016). Uvulifera, a new generic name for Coccobotrys *(Chaetophoraceae)*. Notulae Algarum. 5, 1–3.

[B61] Mostaert A. S. Giordani C. Crockett R. Karsten U. Schumann R. Jarvis S. P. (2009). Characterisation of amyloid nanostructures in the natural adhesive of unicellular subaerial algae. J. Adhes. 85, 465–483. 10.1080/00218460902996366

[B62] Nakada T. Nozaki H. Pröschold T. (2008). Molecular phylogeny, ultrastructure, and taxonomic revision of Chlorogonium (Chlorophyta): emendation of Chlorogonium and description of Gungnir gen. nov. and Rusalka gen. nov. J. Phycol. 44, 751–760. 10.1111/j.1529-8817.2008.00525.x 27041433

[B63] Neustupa J. Němcová Y. Eliáš M. Škaloud P. (2009). Kalinella bambusicola gen. et sp. nov. (Trebouxiophyceae, Chlorophyta), a novel coccoid Chlorella-like subaerial alga from Southeast Asia. Phycol. Res. 57, 159–169. 10.1111/j.1440-1835.2009.00534.x

[B64] Ohtani S. Akiyama M. Kanda H. (1991). Analysis of Antarctic soil algae by the direct observation using the contact slide method. Antarct. Rec. 35, 285–295.

[B65] Pfaff S. Borchhardt N. Boy J. Karsten U. Gustavs L. (2016). Desiccation tolerance and growth-temperature requirements of Coccomyxa (Trebouxiophyceae, Chlorophyta) strains from Antarctic biological soil crusts. Algol. Stud. 151–152, 3–19. 10.1127/algol_stud/2016/0245

[B66] Pröschold T. (2001). Molecular phylogeny and taxonomic revision of chlamydomonas (chlorophyta). I. emendation of chlamydomonas ehrenberg and chloromonas gobi, and description of oogamochlamys gen. nov. and lobochlamys gen. nov. Protist 152, 265–300. 10.1078/1434-4610-00068 11822658

[B67] Pushkareva E. Johansen J. R. Elster J. (2016). A review of the ecology, ecophysiology and biodiversity of microalgae in Arctic soil crusts. Polar Biol. 39, 2227–2240. 10.1007/s00300-016-1902-5

[B68] Rindi F. Mikhailyuk T. I. Sluiman H. J. Friedl T. López-Bautista J. M. (2011). Phylogenetic relationships in Interfilum and Klebsormidium (Klebsormidiophyceae, Streptophyta). Mol. Phylogenet. Evol. 58, 218–231. 10.1016/j.ympev.2010.11.030 21145975

[B69] Ryšánek D. Hrčková K. Škaloud P. (2015). Global ubiquity and local endemism of free-living terrestrial protists: phylogeographic assessment of the streptophyte alga *Klebsormidium*. Environ. Microbiol. 17, 689–698. 10.1111/1462-2920.12501 24803402

[B70] Schulz K. Mikhailyuk T. Dreßler M. Leinweber P. Karsten U. (2016). Biological soil crusts from coastal dunes at the baltic sea: cyanobacterial and algal biodiversity and related soil properties. Microb. Ecol. 71, 178–193. 10.1007/s00248-015-0691-7 26507846

[B71] Sørensen T. (1948). A method of establishing groups of equal amplitude in plant sociology based on similarity of species and its application to analyses of the vegetation on Danish commons. Biol. Skr. K. Danske Vidensk. Selsk. 5, 1–34.

[B72] Starr R. C. Zeikus J. A. (1993). UTEX – the culture collection of algae at the University of Texas at Austin 1993 list of cultures. J. Phycol. 29, 1–106. 10.1111/j.0022-3646.1993.00001.x

[B73] Tedrow J. C. (1977). Soils of the Polar Landscapes. New Brunswick: Rutgers University Press.

[B74] Tschaikner A. Gärtner G. Kofler W. (2008). Coelastrella aeroterrestrica sp. nov. (Chlorophyta, Scenedesmoideae) – a new, obviously often overlooked aeroterrestrial species. Arch. Hydrobiol. Suppl. Algol. Stud. 128, 11–20. 10.1127/1864-1318/2008/0128-0011

[B75] Van Den Ancker J. A. M. Jungerius P. D. (1985). Short Communications: The role of algae in the stabilization of coastal dune blowouts. Earth Surf. Proces. Landforms 10, 189–192. 10.1002/esp.3290100210

[B76] Weber B. Büdel B. Belnap J. (2016). Biological Soil Crusts: An Organizing Principle in Drylands. Cham: Springer International Publishing.

[B77] Whittaker R. H. (1960). Vegetation of the Siskiyou Mountains, Oregon and California. Ecol. Mono. 30, 279–338. 10.2307/1943563

[B78] Whittaker R. H. (1972). Evolution and measurement of species diversity. Taxon 21, 213–251. 10.2307/1218190

[B79] Williams L. Borchhardt N. Colesie C. Baum C. Komsic-Buchmann K. Rippin M. (2016). Biological soil crusts of Arctic Svalbard and of Livingston Island, Antarctica. Polar Biol. 40, 399–411. 10.1007/s00300-016-1967-1

[B80] Wu Y. Rao B. Wu P. Liu Y. Li G. Li D. (2013). Development of artificially induced biological soil crusts in fields and their effects on top soil. Plant Soil. 370, 115–124. 10.1007/s11104-013-1611-6

[B81] Zhang B. Zhang Y. Zhao J. Wu N. Chen R. Zhang J. (2009). Microalgal species variation at different successional stages in biological soil crusts of the Gurbantunggut Desert, Northwestern China. Biol. Fertil. Soils 45, 539–547. 10.1007/s00374-009-0364-0

[B82] Zidarova R. (2008). Algae from Livingston Island (S Shetland Islands): a checklist. Phytolog. Balc. 14, 19–35.

