# Supplementary material for: Biological Soil Crusts of Arctic Svalbard—Water Availability as Potential Controlling Factor for Microalgal Biodiversity
Source: Front Microbiol. 2017 Aug 8;8:1485. doi: 10.3389/fmicb.2017.01485 (PMC5550688; doi:10.3389/fmicb.2017.01485)
Supplement: Table S1 — Algae catalog. Species list of algae and summary of several information about every identified species. x: Presence of the species, black arrow: nucleus, white arrow: pyrenoid, gray arrow: stigma, bar: 10 μm. [file Table1.PDF]

Table S1: Algae catalogue. Species list of algae and summary of several information about every identified species. x: presence of the species, black arrow: nucleus, white arrow: pyrenoid, grey arrow: stigma, bar: 10 µm

species	former name	light micrograph	drawing	reference	NA	LB	isolate number
**Chlorophyceae**							
Bracteacoccus aggregatus		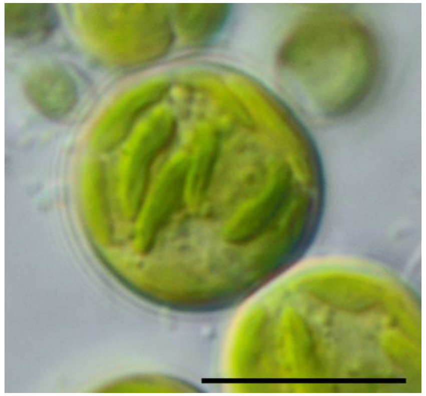 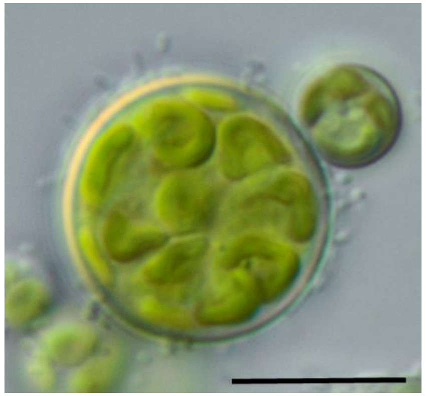		Fucikova, K., Flechtner, V. R. and Lewis, L. A. (2012) Ettl, E. and Gärtner, G. (2014)	x		

Bracteacoccus sp.				Ettl, E. and Gärtner, G. (2014)	x		Geo-5a<br>Geo-5b
Chlamydomonas sp.				Ettl, E. and Gärtner, G. (2014)	x	x	ADC-6a<br>ADC-6b<br>ADC-6c<br>Björn-6a
Chlorococcum lobatum			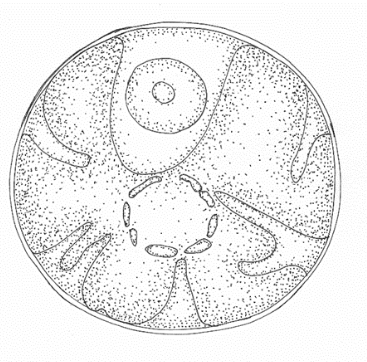	Ettl, E. and Gärtner, G. (2014)		x	
Chlorococcum cf. *minutum*				Ettl, E. and Gärtner, G. (2014)	x		
Chlorococcum sp.				Ettl, E. and Gärtner, G. (2014)	x		D-S-4a<br>London-4a<br>London-4b<br>London-4c

Chloromonas sp.		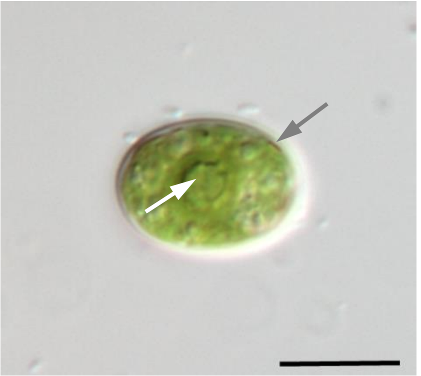		Ettl, E. and Gärtner, G. (2014)	x	x	ADC-8a
Chromochloris zofingiensis	*Muriella zofingiensis, Bracteacoccus minutus*	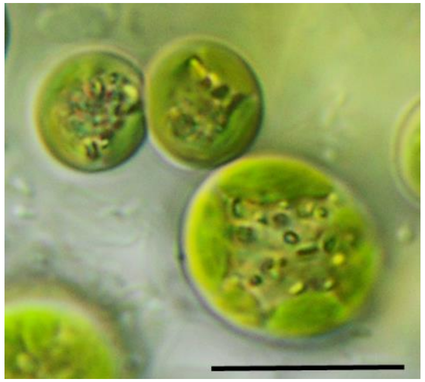		Fucikova, K. and Lewis, L. A. (2012)<br>Ettl, E. and Gärtner, G. (2014)	x		
Coelastrella aeroterrestrica		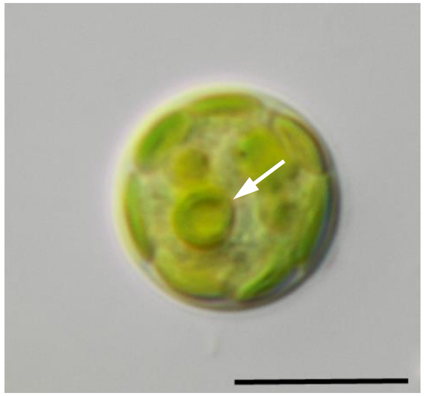	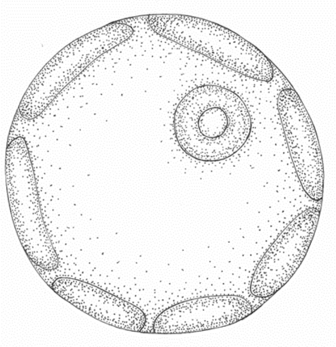	Tschaikner, A., Gärtner, G. and Kofler, W. (2008)	x	x	

Coelastrella rubescens	*Scotiellopsis rubescens*	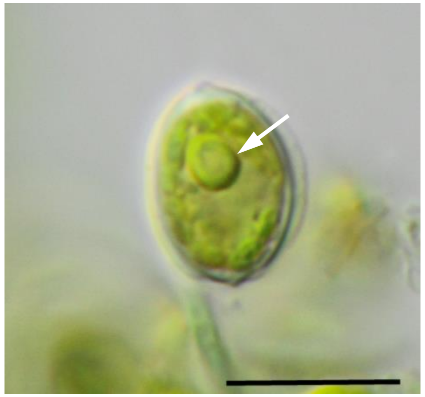 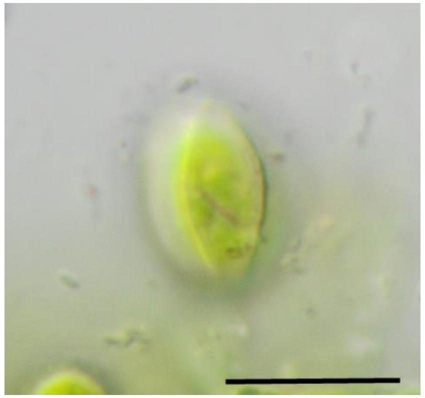	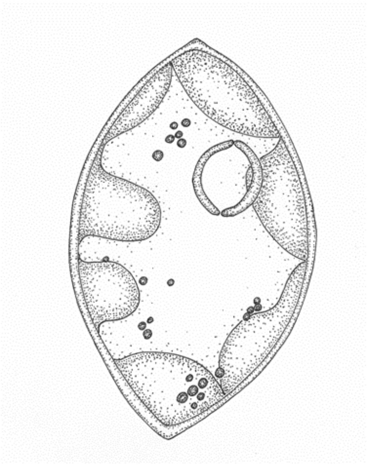	Kaufnerova, V. and Elias, M. (2013)<br>Ettl, E. and Gärtner, G. (2014)	x	x	
Coelastrella cf. *rubescens*				Kaufnerova, V. and Elias, M. (2013)<br>Ettl, E. and Gärtner, G. (2014)	x	x	
Coelastrella sp.	*Scotiellopsis* sp.			Kaufnerova, V. and Elias, M. (2013)		x	

				Ettl, E. and Gärtner, G. (2014)			
Coenobotrys cf. *gloeobotrydiformis*	*Coccomyxa gloeobotrydiformis*	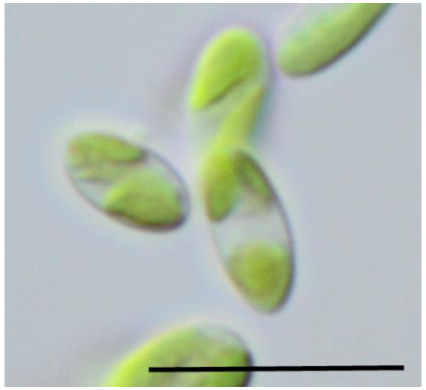		Kostikov, I. et al. (2002)<br><br>Ettl, E. and Gärtner, G. (2014)		x	
Coenochloris sp.	*Radiococcus* sp.			Kostikov, I. et al. (2002)<br><br>Ettl, E. and Gärtner, G. (2014)	x		
Coenocystis oleifera var. *antarctica*	*Sphaerocystis oleifera* var. *antarctica*	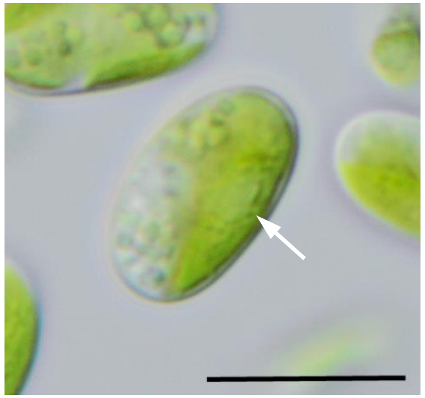	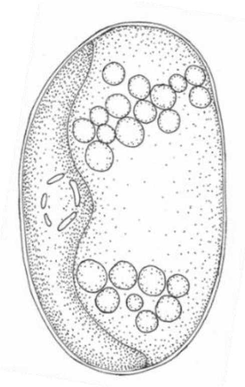	Ettl, E. and Gärtner, G. (2014)	x	x	

Desmodesmus abundans	*Chlorella fusca*	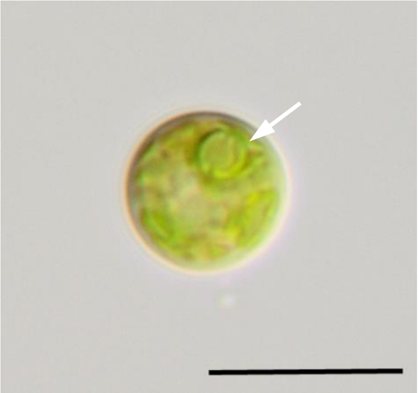 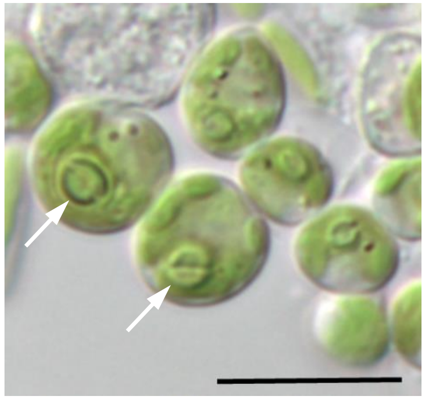		Kessler, E. et al. (1997)<br><br>Ettl, E. and Gärtner, G. (2014)	x	x	
Fasciculochloris cf. *boldii*		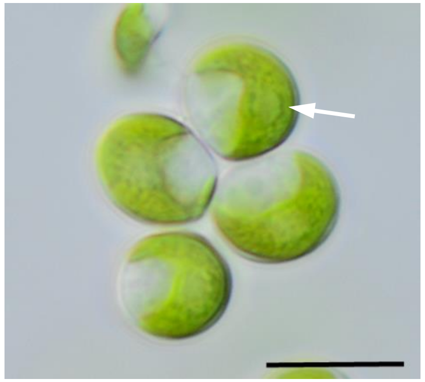		Ettl, E. and Gärtner, G. (2014)	x		

Gloeocystis sp.				Ettl, E. and Gärtner, G. (2014)	x	x	EiE-2a
Gungnir mantoniae	*Chlamydomonas mantonii*	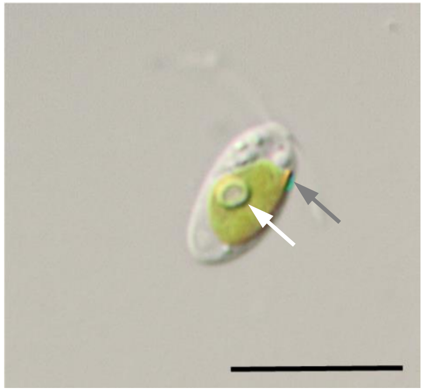 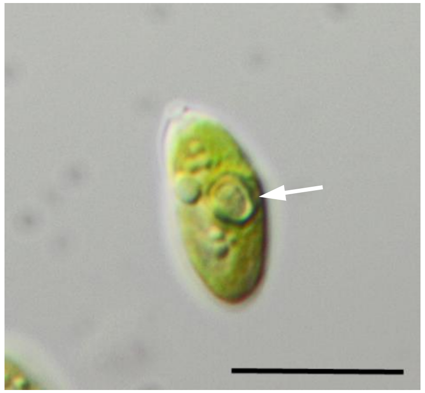		Nakada, T., Nozaki, H., and Pröschold, T. (2008)<br><br>Ettl, E. and Gärtner, G. (2014)	x		SH-Kj-38a

Heterotetracystis akinetos		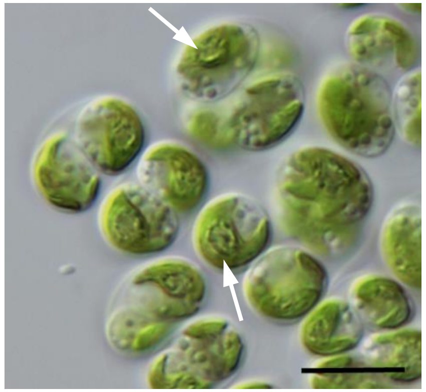		Ettl, E. and Gärtner, G. (2014)		x	Berg-3a
Heterotetracystis intermedia			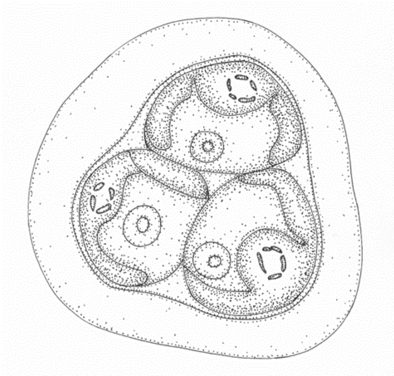	Ettl, E. and Gärtner, G. (2014)	x		

Hormotilopsis sp.		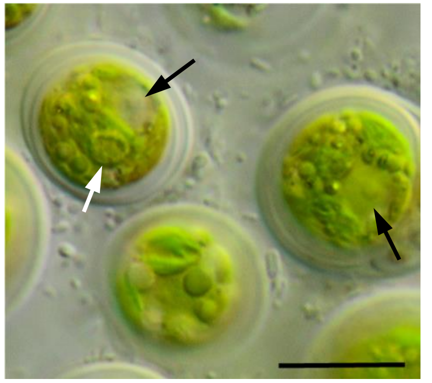 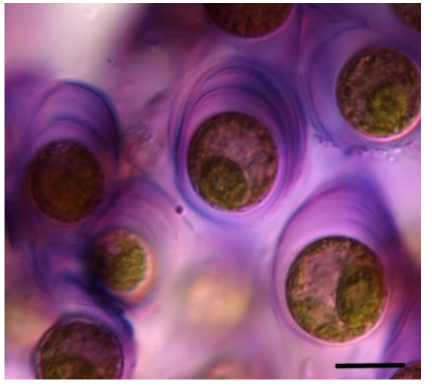		Ettl, E. and Gärtner, G. (2014)		x	
Lobochlamys cf. *culleus*	*Chlamydomonas culleus*		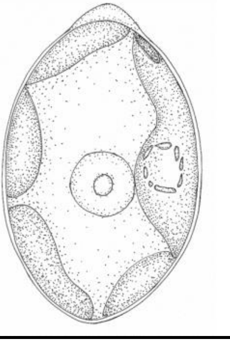	Pröschold, T. et al. (2001) Ettl, E. and Gärtner, G., (2014)	x	x	
Lobochlamys sp.				Pröschold, T. et al. (2001)		x	

Macrochloris cohaerens		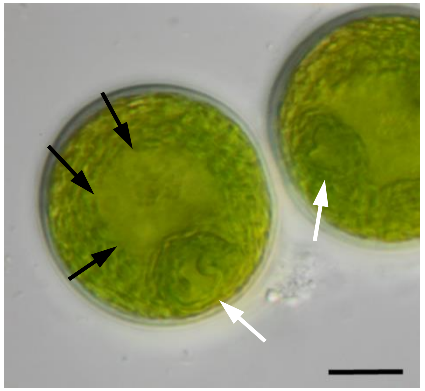	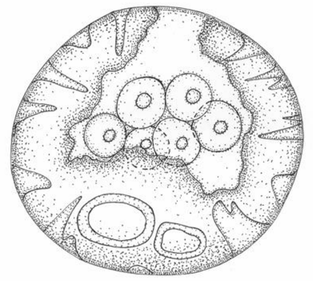	Ettl, E. and Gärtner, G., (2014)	x		NA-18aII
Mychonastes homosphaera	*Chlorella homosphaera, Chlorella miniata, Chlorella minutissima*	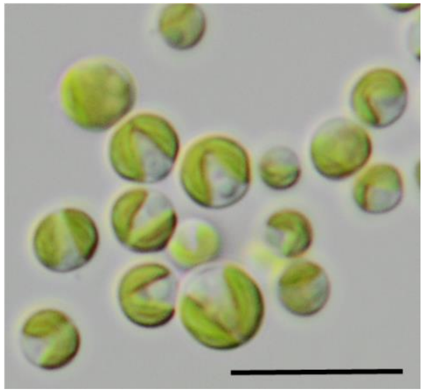		Huss, V. et al. (1999)<br><br>Bock, C. et al. (2011)<br><br>Ettl, E. and Gärtner, G. (2014)	x	x	D-S-34a<br>Hinter-34a<br>Hinter-34b
Pseudodictyochloris multinucleata		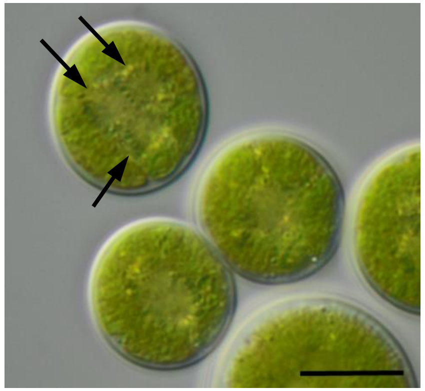		Ettl, E. and Gärtner, G. (2014)	x	x	

Sporotetras polydermatica	*Gloeocystis polydermatica*	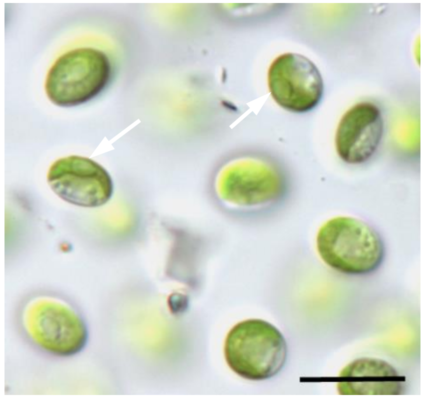	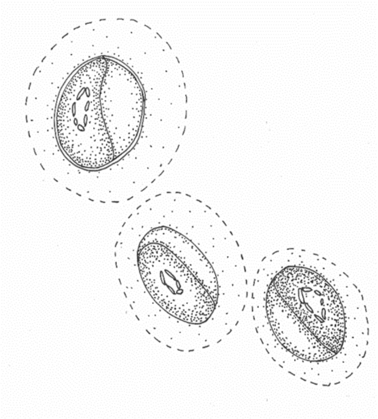	Kostikov, I. et al. (2002)<br><br>Ettl, E. and Gärtner, G. (2014)	x	x	
Tetracystis cf. *fissurata*			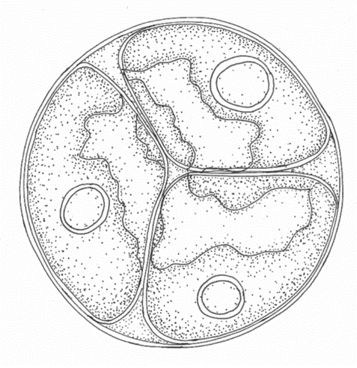	Ettl, E. and Gärtner, G. (2014)	x		NA-33a
Tetracystis sp.				Ettl, E. and Gärtner, G. (2014)	x	x	London-27a
Uvulifera sp.	*Coccobotrys* sp.			Molinari-Novoa, E. A. (2016)<br><br>Ettl, E. and Gärtner, G. (2014)	x		

Trebouxiophyceae							
Chlorella chlorelloides	*Dictyosphaerium chlorelloides*	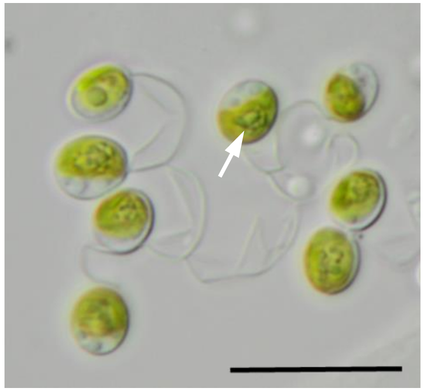 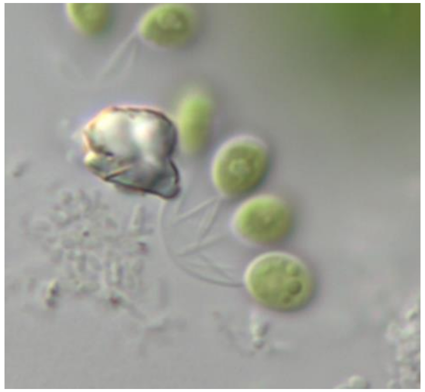		Bock, C. et al. (2011)<br><br>Ettl, E. and Gärtner, G. (2014)		x	
Chlorella vulgaris		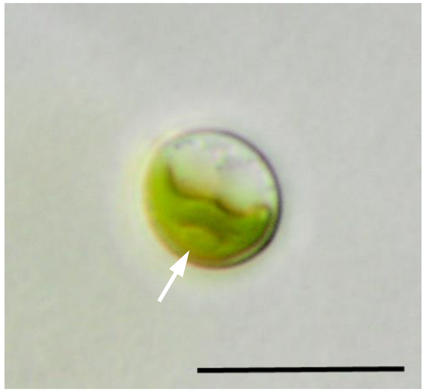		Ettl, E. and Gärtner, G. (2014)	x	x	SH-Kj-39a

Chlorella cf. *vulgaris*				Ettl, E. and Gärtner, G. (2014)	x	x	
Chlorella sp.				Ettl, E. and Gärtner, G. (2014)	x	x	London-35a London-35b
Chloroidium ellipsoideum	*Chlorella ellipsoidea*	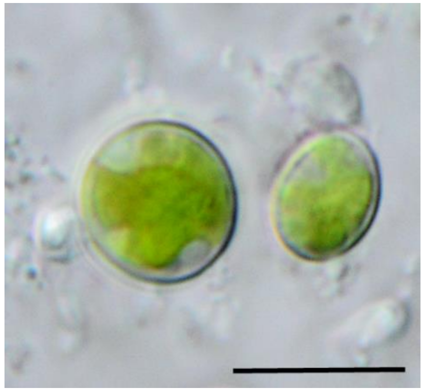		Darienko, T. et al. (2010) Ettl, E. and Gärtner, G. (2014)	x	x	
Chloroidium sp.		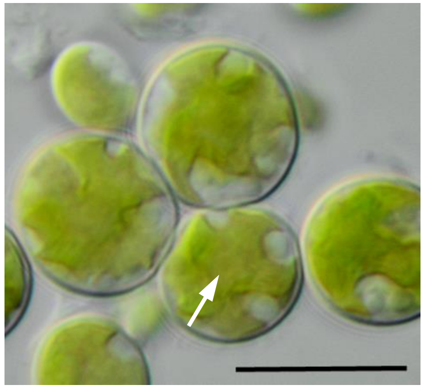		Darienko, T. et al. (2010)	x		NA-31aII

Coccomyxa simplex		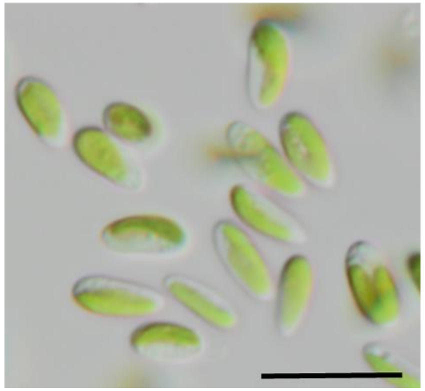		Ettl, E. and Gärtner, G. (2014)<br><br>Darienko, T. et al. (2015)<br><br>Malavasi, V. et al. (2016)	x	x	
Coccomyxa subglobosa				Ettl, E. and Gärtner, G. (2014)<br><br>Darienko, T. et al. (2015)<br><br>Malavasi, V. et al. (2016)	x		
Coccomyxa sp.				Ettl, E. and Gärtner, G. (2014)<br><br>Darienko, T. et al. (2015)<br><br>Malavasi, V. et al. (2016)	x	x	

Desmococcus olivaceus		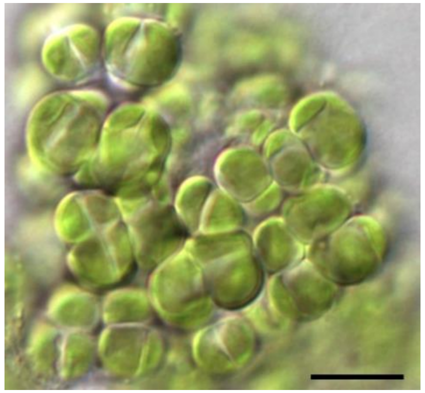	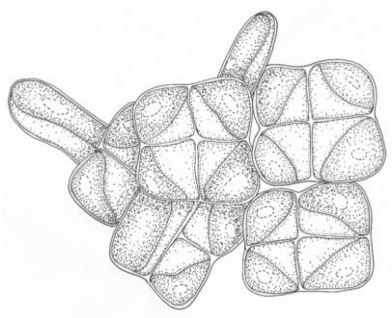	Ettl, E. and Gärtner, G. (2014)	x	x	EiE-7a NA-10aII
Desmococcus sp.				Ettl, E. and Gärtner, G. (2014)	x		
Dictyosphaerium dichotomum		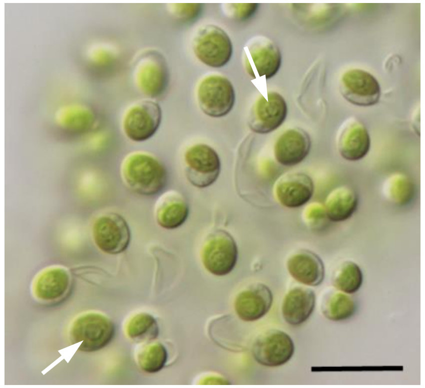		Bock, C., Pröschold, and T., Krienitz, L. (2011)<br><br>Ettl, E. and Gärtner, G. (2014)		x	

Elliptochloris bilobata		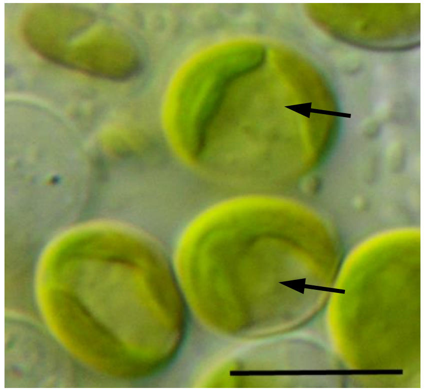		Ettl, E. and Gärtner, G. (2014)	x	x	
Elliptochloris subsphaerica		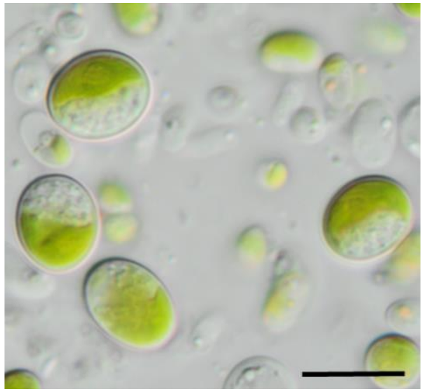		Darienko, T. et al. (2016)	x		
Elliptochloris sp.				Ettl, E. and Gärtner, G. (2014)	x		
Gloeotila scopulina				Ettl, E. and Gärtner, G. (2014)	x		
Heterochlorella luteoviridis	*Chlorella luteoviridis*			Neustupa, J. et al. (2009)	x		

				Ettl, E. and Gärtner, G. (2014)			
Koliella sempervirens	*Raphidonema sempervirens*	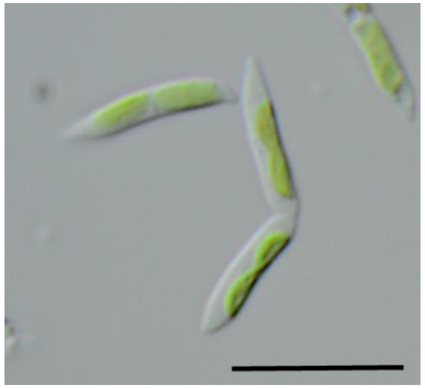	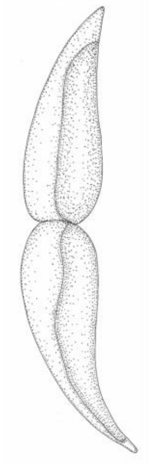	Ettl, E. and Gärtner, G. (2014)	x		NA-23aII
Muriella terrestris		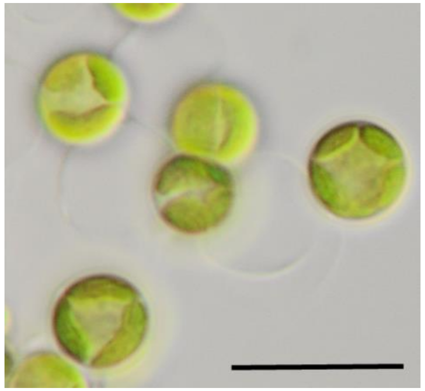		Ettl, E. and Gärtner, G. (2014)	x		London-36a
Muriella sp.				Ettl, E. and Gärtner, G. (2014)	x		D-S-30a<br>D-S-30b<br>D-S-30c
Muriella sp. I (Broady)				Ettl, E. and Gärtner, G. (2014)	x		

Myrmecia bisecta		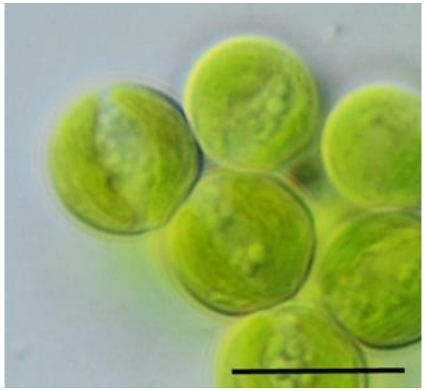	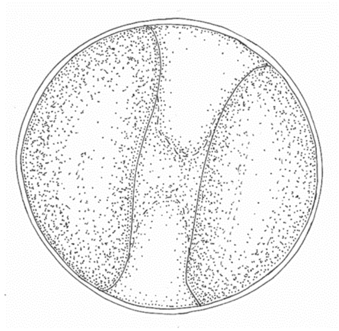	Ettl, E. and Gärtner, G. (2014)	x		
Myrmecia sp.				Ettl, E. and Gärtner, G. (2014)	x		
Neocytis cf. *brevis*	*Coccomyxa brevis*, *Nephrodiella brevis*	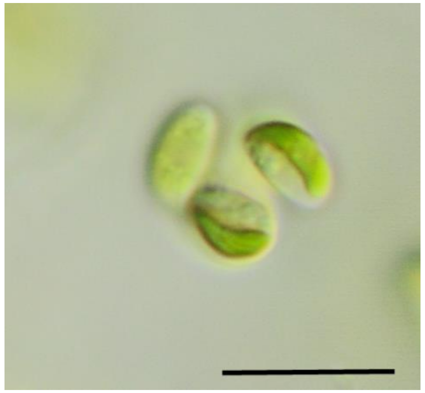		Elias, M. et al. (2013)<br><br>Ettl, E. and Gärtner, G. (2014)	x	x	Börn-29a
Neocystis sp.				Elias, M. et al. (2013)<br><br>Ettl, E. and Gärtner, G. (2014)	x		

Parachlorella cf. *kessleri*	*Chlorella kessleri*			Krienitz, L. et al. (2004)<br>Ettl, E. and Gärtner, G. (2014)		x	
Pseudochlorella cf. *signiensis*	*Pabia signiensis*	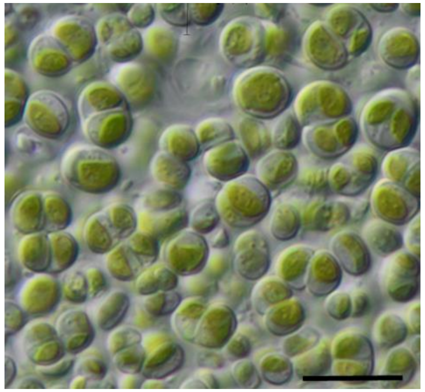		Darienko, T. et al. (2016)		x	
Stichococcus allas		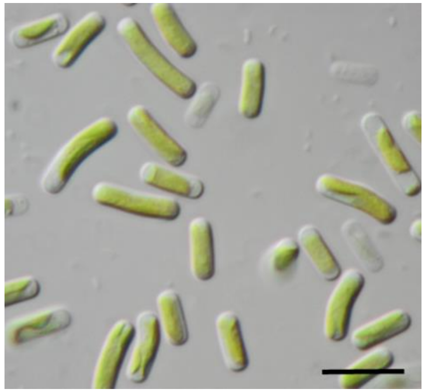		Ettl, E. and Gärtner, G. (2014)	x	x	
Stichococcus cf. *allas*				Ettl, E. and Gärtner, G. (2014)	x	x	

Stichococcus bacillaris		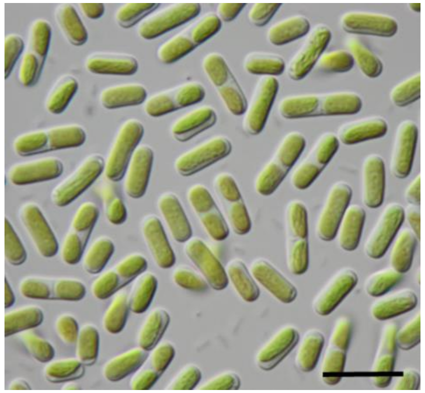		Ettl, E. and Gärtner, G. (2014)	x	x	London-19a NA-19aII
Stichococcus chlorelloides		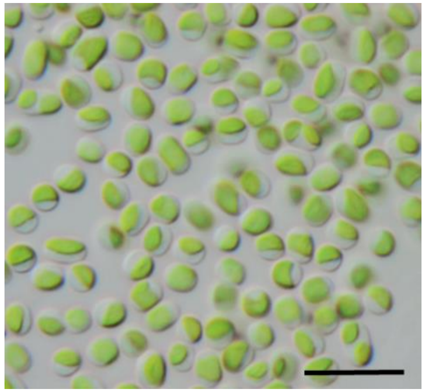		Ettl, E. and Gärtner, G. (2014)	x	x	
Stichococcus exiguus		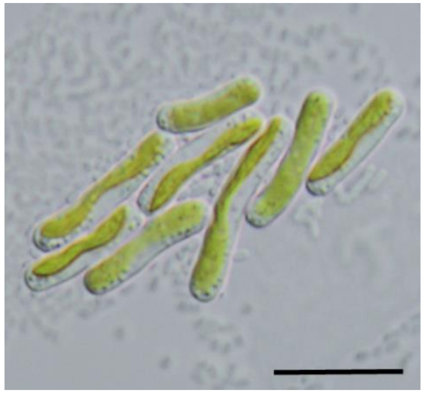	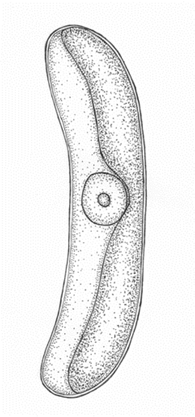	Ettl, E. and Gärtner, G. (2014)	x		NA-37a

Stichococcus cf. *exiguus*				Ettl, E. and Gärtner, G. (2014)	x		
Stichococcus minutus		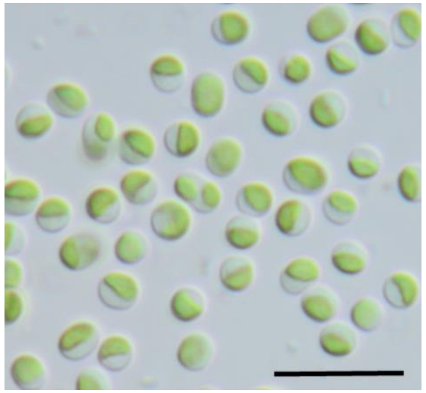		Ettl, E. and Gärtner, G. (2014)	x	x	
Stichococcus cf. *minutus*				Ettl, E. and Gärtner, G. (2014)	x		
Stichococcus sp.				Ettl, E. and Gärtner, G. (2014)	x	x	AD-24a<br>ADC-24a<br>Björn-24a<br>EiE-24a<br>NA-24a
**Klebsormidiophyceae**							
Interfilum cf. *massjukiae*				Mikhailyuk, T. et al. (2008)<br><br>Rindi, F. et al. (2011)<br><br>Karsten, U., Herburger,		x	

				K. and Holzinger, A. (2014)			
Interfilum sp.				Mikhailyuk, T. et al. (2008)<br><br>Rindi, F. et al. (2011)<br><br>Ettl, E. and Gärtner, G. (2014)<br><br>Karsten, U., Herburger, K. and Holzinger, A. (2014)	x	x	ADC-12a<br>D-S-12a<br>D-S-12b<br>D-S-12c<br>Geo-12a
Klebsormidium cf. *crenulatum*		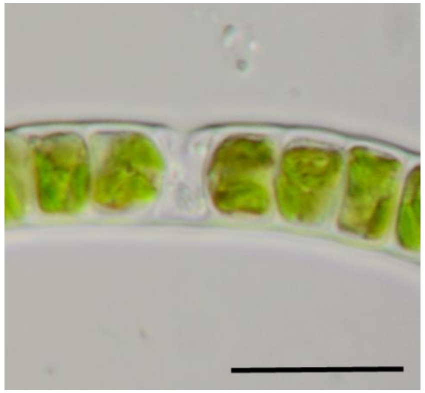		Rindi, F. et al. (2011)<br><br>Ettl, E. and Gärtner, G. (2014)<br><br>Mikhailyuk, T. et al. (2014)	x		Björn-11a<br>Hinter-11aII

Klebsormidium cf. *dissectum*		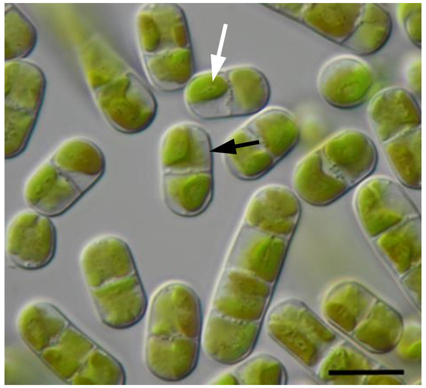 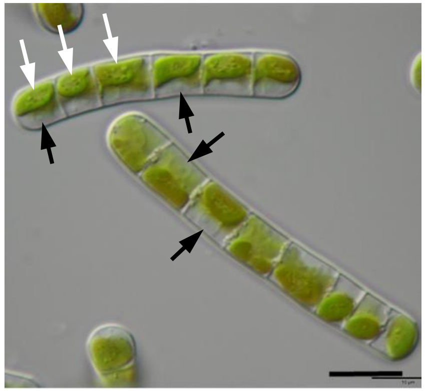		Rindi, F. et al. (2011)<br><br>Ettl, E. and Gärtner, G. (2014)<br><br>Mikhailyuk, T. et al. (2014)	x	x	EiE-15a Hinter-15a
Klebsormidium cf. *flaccidum*		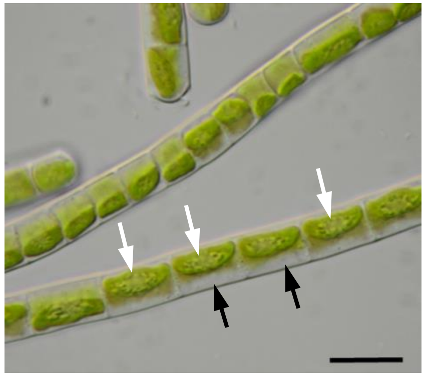		Ettl, E. and Gärtner, G. (2014)<br><br>Mikhailyuk, T. et al. (2014)	x	x	
Klebsormidium cf. klebsii				Rindi, F. et al., (2011)	x		

				Ettl, E. and Gärtner, G. (2014)			
Klebsormidium cf. *montanum*		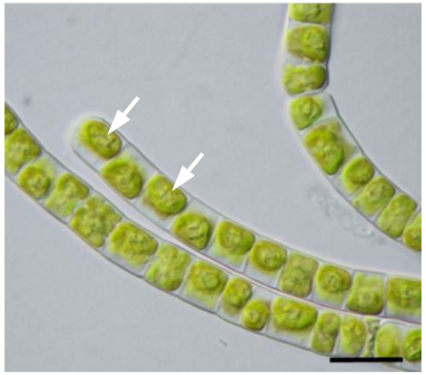		Rindi, F. et al. (2011)<br><br>Ettl, E. and Gärtner, G., (2014)	x	x	
Klebsormidium cf. *nitens*				Ettl, E. and Gärtner, G. (2014)<br><br>Mikhailyuk, T. et al. (2014)	x	x	AD-16a
Klebsormidium cf. *subtile*		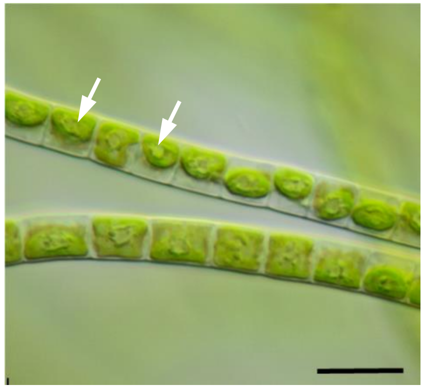		Ettl, E. and Gärtner, G. (2014)		x	AD-14a<br>EiE-14a

Klebsormidium sp.				Ettl, E. and Gärtner, G. (2014)	x	x	AD-17a
Klebsormidium sp. (G-Clade)		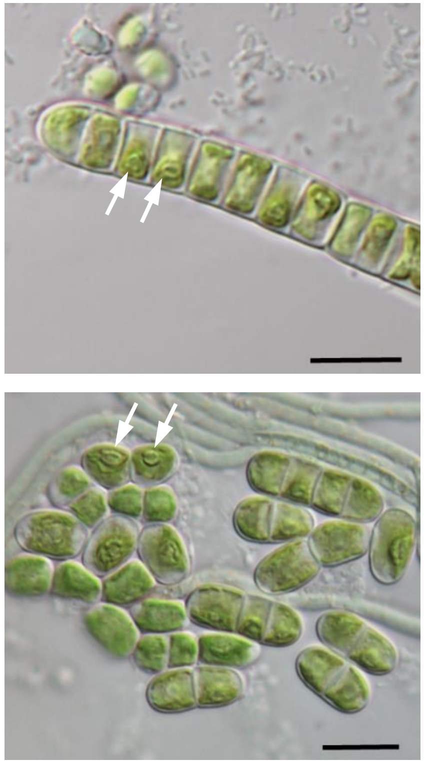	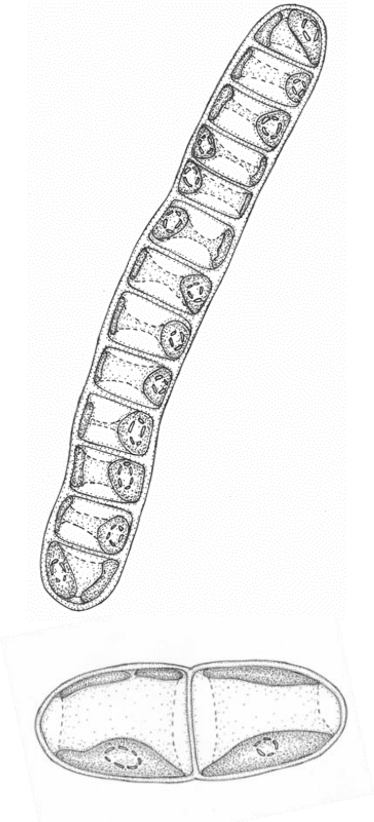	Rindi, F. et al. (2011)<br>Mikhailyuk, T. et al. (2014)	x	x	
**Zygnematophyceae**							
Actinotaenium sp.				Ettl, E. and Gärtner, G. (2014)		x	

Cylindrocystis crassa		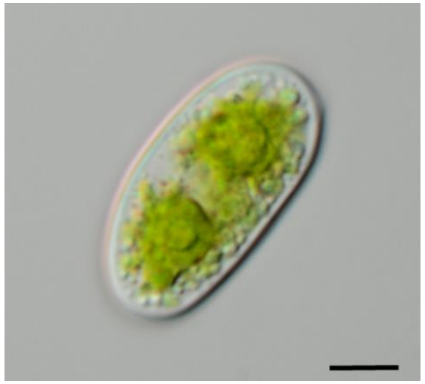		Ettl, E. and Gärtner, G. (2014)	x		
**Eustigmatophyceae**							
Eustigmatos vischeri		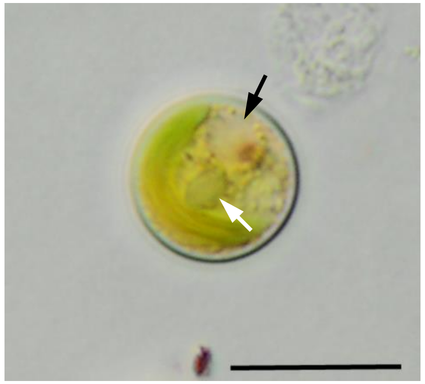		Ettl, E. and Gärtner, G. (2014)	x	x	
Eustigmatos cf. *vischeri*				Ettl, E. and Gärtner, G. (2014)		x	
**Xanthophyceae**							

Botrydiopsis intercedens	*Botrydiopsis alpina*	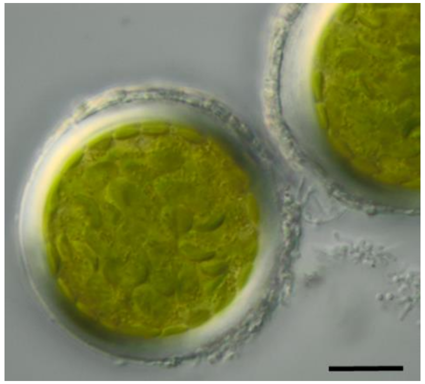 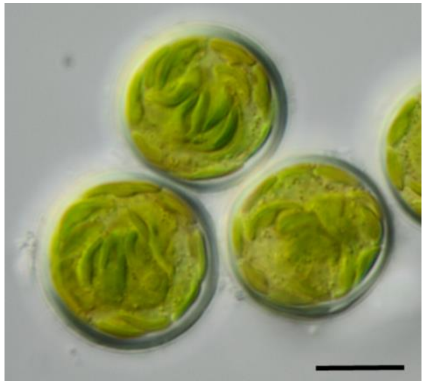 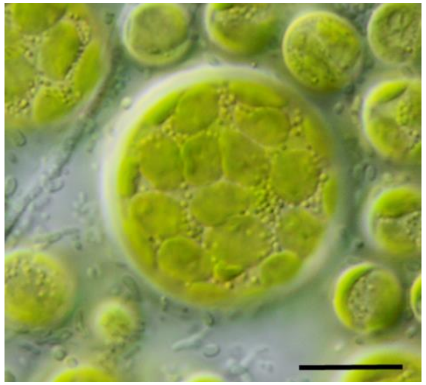		Ettl, E. and Gärtner, G (2014)	x		NA-32aII<br>NA-32bII
Botrydiopsis arhiza				Ettl, E. and Gärtner, G. (2014)	x		London-1a<br>Geo-1a

Botrydiopsis cf. *constricta*				Ettl, E. and Gärtner, G., (2014)	x		
Chlorellidium cf. *tetrabotrys*				Ettl, E. and Gärtner, G. (2014)		x	
Chloridella sp.		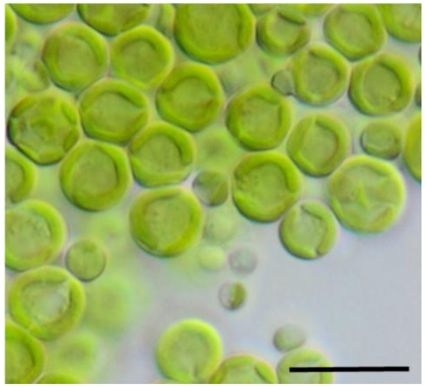 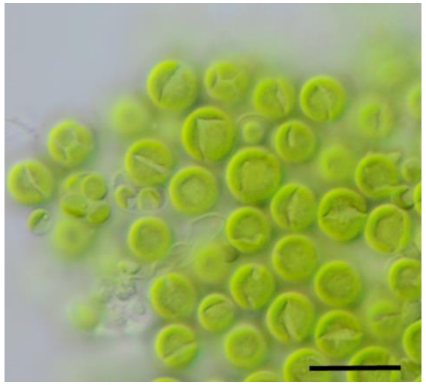		Ettl, E. and Gärtner, G. (2014)	x		

Heterococcus sp.		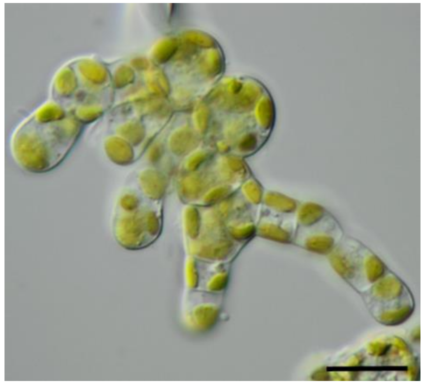		Ettl, E. and Gärtner, G. (2014)	x	x	Björn-9a D-S-9a London-9a London-9b NA-9aII NA-9bII
Monallantus brevicylindrus				Ettl, E. and Gärtner, G. (2014)	x		
Nephrodiella cf. *phaseolus*				Ettl, E. and Gärtner, G. (2014)	x		
Pleurochloris meiringensis		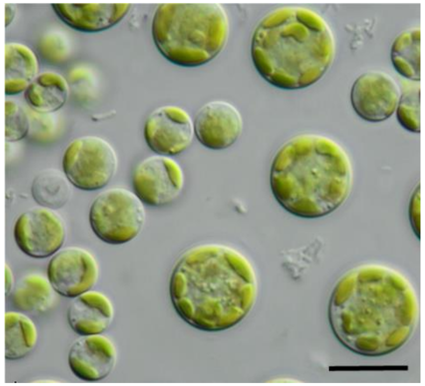		Ettl, E. and Gärtner, G. (2014)	x	x	NA-22aII

Pleurochloris pseudopolychloris		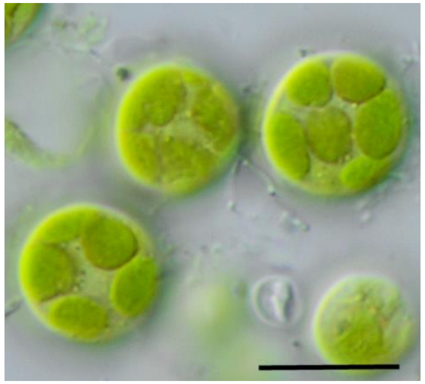		Ettl, E. and Gärtner, G. (2014)	x	x	AD-21a
Pleurochloris polychloris		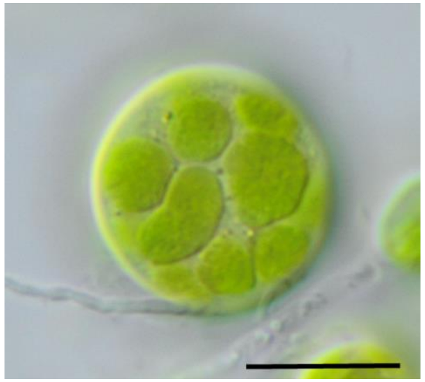		Ettl, E. and Gärtner, G. (2014)	x		

Pleurogaster lunaris		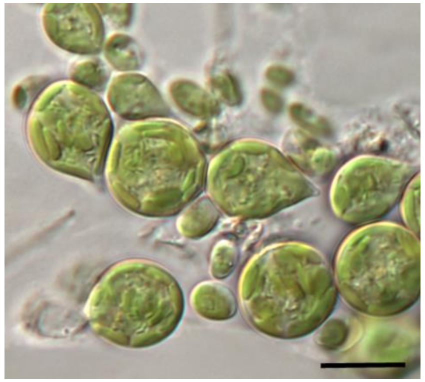 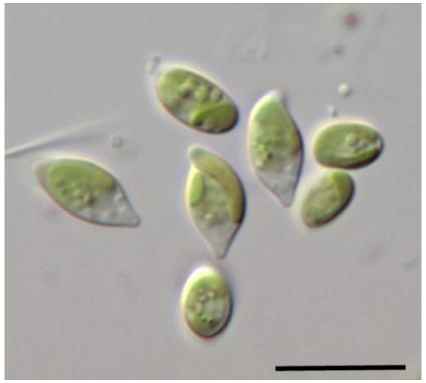	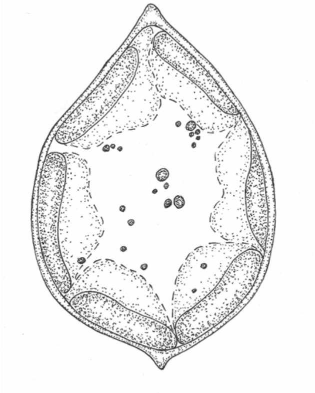 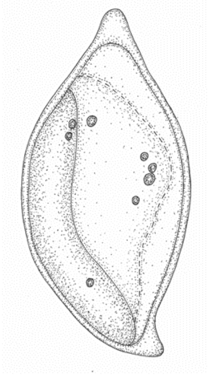	Ettl, E. and Gärtner, G. (2014)	x	x	
Tribonema viride		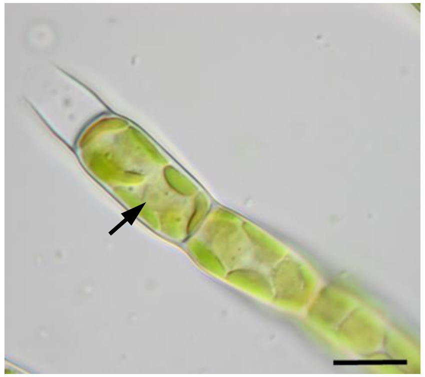		Ettl, E. and Gärtner, G. (2014)	x		

Tribonema vulgare				Ettl, E. and Gärtner, G. (2014)	x		
Tribonema sp.				Ettl, E. and Gärtner, G. (2014)	x		
Xanthonema cf. *debile*				Ettl, E. and Gärtner, G. (2014)	x	x	AD-28a
Xanthonema solidum		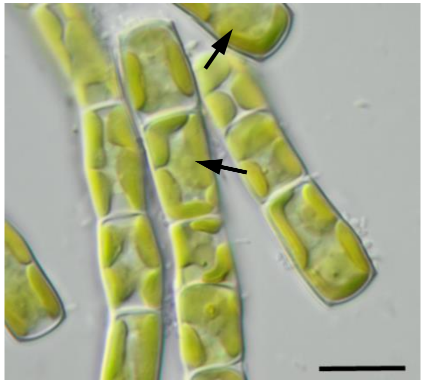		Ettl, E. and Gärtner, G. (2014)	x	x	
Xanthonema cf. *solidum*				Ettl, E. and Gärtner, G. (2014)	x		NA-25aII

Xanthonema exile		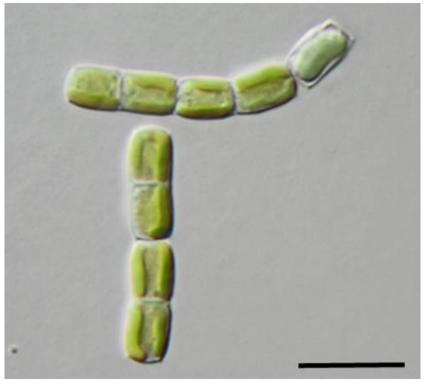		Ettl, E. and Gärtner, G. (2014)	x		
Xanthonema sp.				Ettl, E. and Gärtner, G. (2014)	x	x	ADC-26a NA-26aII
