# Supplementary figures and images for: Biological Soil Crusts of Arctic Svalbard—Water Availability as Potential Controlling Factor for Microalgal Biodiversity
Source: Front Microbiol. 2017 Aug 8;8:1485. doi: 10.3389/fmicb.2017.01485 (PMC5550688; doi:10.3389/fmicb.2017.01485)

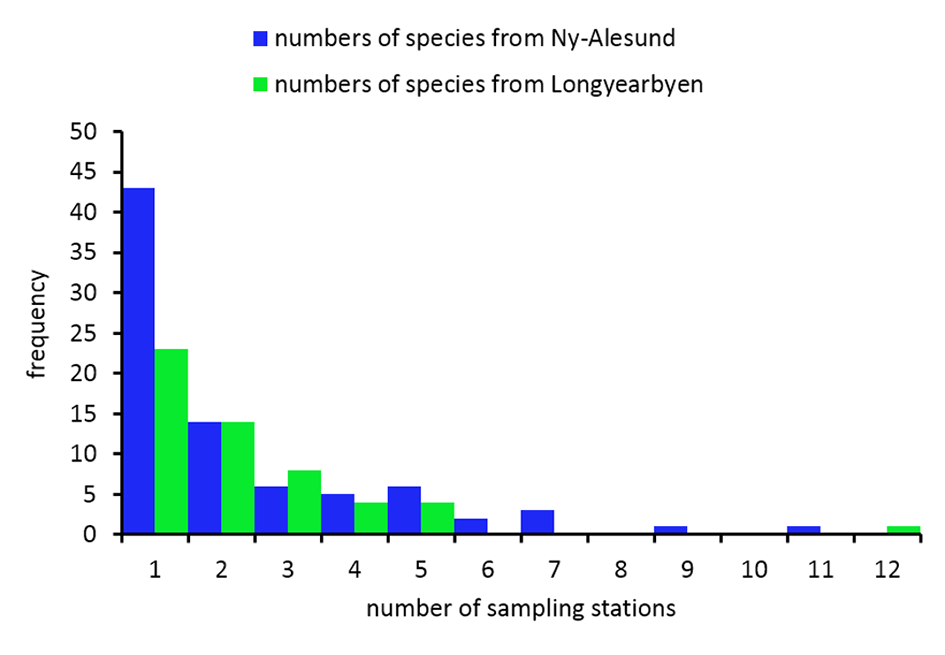

Supplement: Figure S1 — Frequency histogram. Number of species which were found in different numbers of sampling stations on Svalbard. [file Image1.TIF]
